# Metabolic pathways for the whole community

**DOI:** 10.1186/1471-2164-15-619

**Published:** 2014-07-22

**Authors:** Niels W Hanson, Kishori M Konwar, Alyse K Hawley, Tomer Altman, Peter D Karp, Steven J Hallam

**Affiliations:** Graduate Program in Bioinformatics, University of British Columbia, Genome Sciences Centre, 100-570 West 7th Avenue, Vancouver, British Columbia V5Z 4S6 Canada; Department of Microbiology & Immunology, University of British Columbia, 2552-2350 Health Sciences Mall, Vancouver, British Columbia V6T 1Z3 Canada; Biomedical Informatics Training Program, Stanford University, MSOB, 1265 Welch Road, X-215 MC 5479 Stanford, CA 94305-5479 USA; Bioinformatics Research Group, SRI International, 333 Ravenswood Avenue, Menlo Park, CA 94025-3493 USA

## Abstract

**Background:**

A convergence of high-throughput sequencing and computational power is transforming biology into information science. Despite these technological advances, converting bits and bytes of sequence information into meaningful insights remains a challenging enterprise. Biological systems operate on multiple hierarchical levels from genomes to biomes. Holistic understanding of biological systems requires agile software tools that permit comparative analyses across multiple information levels (DNA, RNA, protein, and metabolites) to identify emergent properties, diagnose system states, or predict responses to environmental change.

**Results:**

Here we adopt the MetaPathways annotation and analysis pipeline and Pathway Tools to construct environmental pathway/genome databases (ePGDBs) that describe microbial community metabolism using MetaCyc, a highly curated database of metabolic pathways and components covering all domains of life. We evaluate Pathway Tools’ performance on three datasets with different complexity and coding potential, including simulated metagenomes, a symbiotic system, and the Hawaii Ocean Time-series. We define accuracy and sensitivity relationships between read length, coverage and pathway recovery and evaluate the impact of taxonomic pruning on ePGDB construction and interpretation. Resulting ePGDBs provide interactive metabolic maps, predict emergent metabolic pathways associated with biosynthesis and energy production and differentiate between genomic potential and phenotypic expression across defined environmental gradients.

**Conclusions:**

This multi-tiered analysis provides the user community with specific operating guidelines, performance metrics and prediction hazards for more reliable ePGDB construction and interpretation. Moreover, it demonstrates the power of Pathway Tools in predicting metabolic interactions in natural and engineered ecosystems.

**Electronic supplementary material:**

The online version of this article (doi:10.1186/1471-2164-15-619) contains supplementary material, which is available to authorized users.

## Background

Community interactions between uncultivated microorganisms give rise to dynamic metabolic networks integral to ecosystem function and global scale biogeochemical cycles [1]. Metagenomics bridges the “cultivation gap” through plurality or single-cell sequencing by providing direct and quantitative insight into microbial community structure and function [2, 3]. Although, new technologies are rapidly expanding our capacity to chart microbial sequence space, persistent computational and analytical bottlenecks impede comparative analyses across multiple information levels (DNA, RNA, protein and metabolites) [4, 5]. This in turn limits our ability to convert the genetic potential and phenotypic expression of microbial communities into predictive insights and technological or therapeutic innovations.

Functional genes operate within the structure of metabolic pathways and reactions that define metabolic networks. Despite this fact, few metagenomic studies use pathway-centric approaches to predict microbial community interaction networks based on known biochemical rules. Recently, algorithms for pathway prediction and metabolic flux have been developed for environmental sequence information including the Human Microbiome Project Unified Metabolic Analysis Network (HUMAnN) and Predicted Relative Metabolic Turnover (PRMT). HUMAnN uses an integer optimization algorithm that conservatively computes a parsimonious minimum set of reactions along KEGG pathways based on pathway presence, absence or completion [6, 7]. PRMT infers metabolic flux based on normalized enzyme activity counts mapped to KEGG pathways across multiple metagenomes [8]. Because KEGG pathways are coarse and do not discriminate between pathway variants, both modes of analysis have limited metabolic resolution [9]. Moreover, neither HUMAnN nor PRMT provides a coherent structure for exploring and interpreting predicted KEGG pathways.

One alternative to HUMAnN and PRMT is Pathway Tools, a production-quality software environment supporting metabolic inference and flux balance analysis based on the MetaCyc database of metabolic pathways and enzymes representing all domains of life [10–13]. Unlike KEGG or SEED subsystems, MetaCyc emphasizes smaller, evolutionarily conserved or co-regulated units of metabolism and contains the largest collection (over 2000) of experimentally validated metabolic pathways. Extensively commented pathway descriptions, literature citations, and enzyme properties combined within a pathway/genome database (PGDB) provide a coherent structure for exploring and interpreting predicted pathways. Although initially conceived for cellular organisms, recent development of the MetaPathways pipeline extends the PGDB concept to environmental sequence information enabling pathway-centric insights into microbial community structure and function [14, 15].

**Figure 1:**
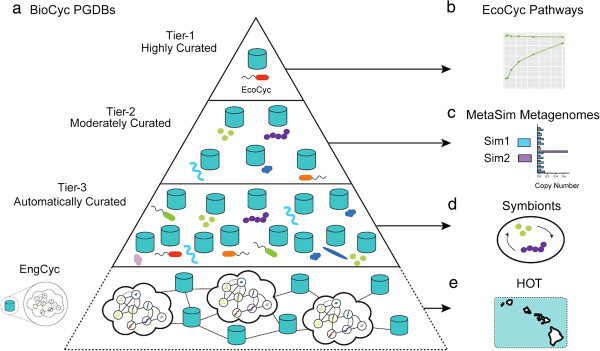
**A multi-tiered approach to ePGDB validation. (a)** In the absence of highly curated and validated datasets, we took inspiration from the curation-tiered structure of available pathway/genome databases within the BioCyc family. **(b/c)** Through *in silico* simulated sequencing experiments on the *E. coli K12* genome and two simulated metagenomes, we evaluated the performance of the PathoLogic algorithm under changing sequence coverage and taxonomic distributions. **(d)** We reanalyzed the genomes of *Candidatus Moranella endobia* and *Candidatus Tremblaya princeps*, two symbiotic taxa with reduced genomes, sharing a number of essential amino acid pathways. **(e)** Finally, we predicted pathways from a previously analyzed paired metagenomic and metatranscriptomic dataset from the Hawaii Ocean Time-series to validate on previously identified pathways and metabolic functions.

Here we provide essential guidelines for generating and interpreting ePGDBs inspired by the multi-tiered structure of BioCyc [16] (Figure 1). We begin with genome and metagenome simulations to assess performance on datasets manifesting different read length, coverage and taxonomic diversity and we develop a weighted taxonomic distance to evaluate concordance between pathways predicted using environmental sequence information and reference pathways in the MetayCyc database. Given these metrics, we demonstrate Pathway Tools’ power to predict emergent metabolism in simulated metagenomes and a previously characterized symbiotic system [17]. Finally, we generate ePGDBs using coupled metagenomic and metatranscriptomic datasets from the Hawaii Ocean Time-series (HOT) to compare and contrast genetic potential and phenotypic expression along defined environmental gradients in the ocean [18–20].

## Results and discussion

### Performance considerations

**Figure 2:**
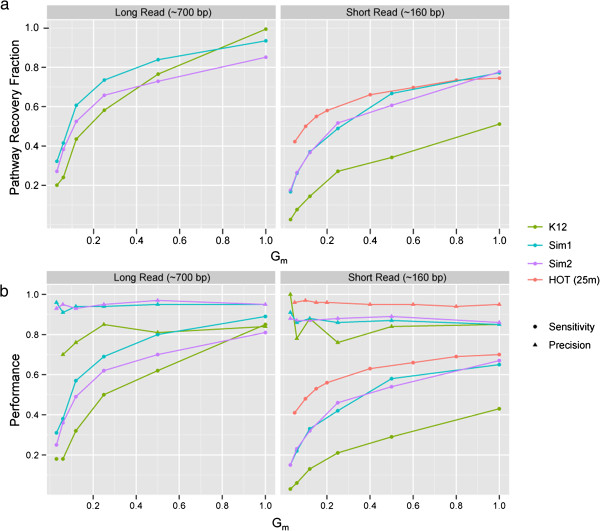
**Analysis on** ***in silico*** **simulated sequencing experiments across different levels of coverage, sequencing lengths, and taxonomic distributions. (a)** Predicted pathway recovery as a fraction of the total pathways predicted from the full genomes. **(b)** Sensitivity (circles) and precision (triangles) of predicted pathways of the *in silico* experiments using the pathways predicted on the full genomes as the gold standard.

Environmental pathway/genome database (ePGDB) construction commences with the MetaPathways automated annotation pipeline using environmental sequence information as input (Materials and Methods). Resulting annotations are used by the PathoLogic algorithm implemented in Pathway Tools to predict metabolic pathways based on multiple criteria including proportion of pathways found, pathway specific enzymatic reactions, and purported taxon-specific pathway distributions. PathoLogic is known to perform well when compared to machine learning methods using the genomes of cellular organisms as input [21]. We previously reported PathoLogic’s performance on combined and incomplete genomes using two simulated metagenomes (Sim1 and Sim2) derived from 10 BioCyc tier-2 PGDBs manifesting different coverage and taxonomic diversity using MetaSim [14, 22]. Simulations on increasing proportions of the total component genome length (G_m_) showed that the performance of pathway recovery based on multiple metrics (F-measure, Matthews Correlation Coefficient, etc.) increased with sequence coverage and sample diversity nearing an asymptote at higher coverage (Figure 2a). This suggests that pathway prediction follows a collector’s curve in which common core pathways accumulate in the early part of the curve followed by less common accessory pathways near the asymptote.

To better constrain pathway recovery and performance in relation to ePGDB construction we compared results of MetaSim experiments using the *Esherichia coli K12 substr. MG1655* genome (basis of the EcoCyc database), Sim1 and Sim2, and a subsampled 25 m metagenome from HOT [19] (Additional file 1: Materials and Methods, Tables S1-S4 and Figure S1). Simulations were performed at progressively larger G_m_ coverage. Consistent with previous observations for Sim1 and Sim2, all experiments showed that pathway recovery percentage and performance sensitivity increased with sequence coverage and sample diversity nearing an asymptote at higher coverage (Figure 2a-b). The absolute values of these patterns were sensitive to read length and likely reflected limits imposed by open reading frame prediction and BLAST/LAST-based annotation. In contrast, performance specificity was high (>85%) regardless of read length, coverage, or taxonomic diversity (Figure 2b). The rate of pathway recovery increased proportionally with increasing sample diversity at lower coverage values, as seen in the reduction of pathway recovery percentage between Sim1, Sim2 and *E. coli* for long read (~700 bp) and between HOT, Sim1/2 and *E. coli* for short read (~160 bp) datasets. Additional performance metrics can be found in Additional file 1: Tables S5–S8. Because PathoLogic performance improves with increasing read length, coverage and sample diversity, sequencing platform selection and use of assembled versus unassembled sequence information should be considered when generating ePGDBs.

When constructing PGDBs for individual genomes PathoLogic uses a process called taxonomic pruning to constrain pathway predictions within a specified taxonomic lineage by taking advantage of the curated ‘taxonomic-range’ associated with a given pathway. For example, if a pathway is found only in plants, it will be difficult to predict this pathway in the genome of a bacterial isolate when using taxonomic pruning. Such a process is intended to reduce false positive predictions in individual genomes [12]; However, microbial communities are composed of diverse and largely uncultivated lineages whose combined metabolic potential and phenotypic expression must be considered both within and between individuals. Thus the taxonomic origin of environmental sequence information is more difficult to ascertain with the same degree of certainty as individual microbial genomes sourced from isolates or single-cells. Indeed, the true taxonomic range of many pathways remains to be constrained given the limited number of isolate genomes and the proclivity for horizontal gene transfer within microbial communities.

In order to evaluate the impact of taxonomic pruning on pathway recovery from environmental sequence information we constructed ePGDBs enabling or disabling taxon-specific pathway distributions (Additional file 1: Table S9). We ran PathoLogic on Sim1/2 and 25 m HOT datasets with the ‘Unclassified sequences’ pruning threshold and without pruning. With taxonomic pruning enabled, long read and short read Sim1 ePGDBs exhibited a reduction of 56% (206 compared to 604) and 61% (194 compared to 499) predicted pathways, respectively. Interestingly, the subsampled 25 m HOT dataset exhibited a 28% reduction (425 compared to 593) in pathway recovery with and without pruning suggesting that increased sample complexity can partially offset taxon specific sensitivity losses. In all cases, the pathways predicted with taxonomic pruning were a subset of pathways predicted without taxonomic pruning. Given these observations we posit that strict taxonomic pruning is inappropriate for ePGDB construction while recognizing potential prediction hazards associated with pathways predicted outside of their expected taxonomic range.

To evaluate concordance between pathways predicted using environmental sequence information and reference pathways in the MetaCyc database we developed a weighted taxonomic distance (WTD) algorithm. The WTD algorithm measures the taxonomic distance between predicted coding DNA sequences (CDS), e.g., BLAST hits from the RefSeq database, and expected taxonomic range for each predicted pathway using the NCBI Taxonomy Database. The NCBI Taxonomy Database is hierarchically structured, and a path between the lowest common ancestor (LCA) of observed CDS annotations and each member of the expected taxonomic range in a pathway can be charted [23], where each path length represents some measure of taxonomic distance e.g. root, cellular organism, domain, phylum/division, class, order, family, genus, species. Steps on the path near the root of the hierarchy define greater evolutionary distances than those near the tips. Thus the WTD algorithm weights steps on the connecting path by a factor of , where *d* is the depth position of a particular taxon in the hierarchy (Additional file 1: Supplementary Note 2). To distinguish between paths descending from the expected taxonomic range and those falling outside the expected taxonomic range, paths descending from an expected taxonomic range have a non-negative distance and paths outside this range have a negative distance. The WTD algorithm gives preference to non-negative distances within expected taxonomic range(s), returning the minimum distance if found. Otherwise the maximum negative distance (i.e., closest to zero) is returned.

When the WTD algorithm was applied to HOT datasets, the taxonomic distribution of predicted pathways generally aligned with the expected taxonomic ranges of MetaCyc Pathways (Additional file 1: Figure S2). Predicted pathways were classified into four categories of taxonomic disagreement based on their WTD: “None” if the WTD was positive, and “Low”, “Medium”, and “High” if less than or equal to zero, based on distance quartiles. A pathway had “Low” taxonomic disagreement if in the upper two quartiles of negative distances (i.e., those closest to zero), “Medium” if in the second quartile, and “High” if in the bottom (i.e., most negative) quartile. Pathways with expected taxonomic ranges affiliated with bacteria and archaea dominated the “None”, “Low”, and “Medium” disagreement classes, while pathways with expected taxonomic ranges affiliated with eukaryotes including “animals”, “fungi”, and “plants” comprised the majority of the “High” disagreement class (Additional file 1: Figure S3). While not excluded from downstream analysis, pathways with distances in the “High” disagreement class are more likely to represent false positives and should be interpreted with care.

### Distributed metabolic pathways

Public good dynamics play an integral role in shaping microbial interactions through distributed networks of metabolite exchange [24]. Such networks promote increased fitness and resilience and may explain the underlying difficulty in cultivating most environmental microorganisms [25–27]. Because ePGDBs are constructed from environmental sequence information, predicted pathways are represented by multiple donor genotypes providing different levels of sequence coverage for each reaction. By comparing pathway recovery for individual reference genomes to pathway recovery for combinations of reference genomes, it becomes formally possible to use Pathway Tools to identify distributed metabolic pathways that emerge between multiple interacting partners. To test this hypothesis, we selected four Tier-2 reference genomes used in simulation experiments and constructed ePGDBs using all possible pair-wise genome combinations (Additional file 1: Table S10). Thirty distributed pathways were identified in pair-wise genome combinations that were not predicted in PGDBs for individual cellular organisms using set-difference analysis (Additional file 1: Table S11). Common and unique reactions associated with distributed pathways could be identified as composite glyphs in the Pathway Tools genome browser (Additional file 1: Figure S4).

**Figure 3:**
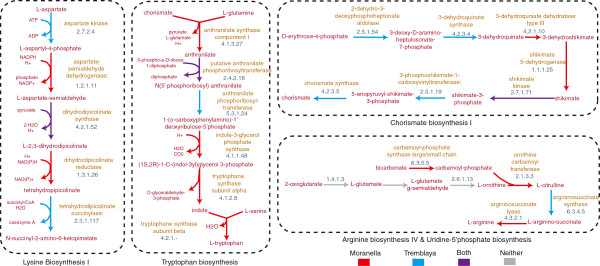
**Examples of emergent amino acid metabolism shared between the** ***Moranella endobia*** **and** ***Tremblaya princeps*** **genomes.** This figure illustrates examples of emergent metabolic pathways predicted between symbiotic prokaryotes *Candidatus Moranella endobia* and *Candidatus Tremblaya princeps*. Enyzmes found in *Moranella* (red), *Tremblaya* (blue), or both taxa (purple) are highlighted in the pathway glyph diagrams, showing patterns of potentially emergent metabolism. A complete description of all amino acid pathways can be found in Additional file 1: Figure S3.

To provide a real world example of distributed metabolic pathway prediction we selected a symbiotic system with known nutritional provisioning requirements. The reduced genomes of *Candidatus Moranella endobia* and *Candidatus Tremblaya princeps* (GenBank NC-015735 and NC-015736), bacterial endosymbionts of the mealybug *Planococcus citri* have been previously described by McCutcheon and colleagues to distribute biosynthetic pathways for essential amino acids in a process known as “inter-pathway complementarity.” Environmental PGDB construction using the combined *Moranella* and *Tremblaya* genomes recovered 43 out of 44 reactions and all 9 distributed amino acid biosynthesis pathways previously reported (Figure 3 and Additional file 1: Figure S5). Given these results, combinatorial ePGDB construction has enormous potential to predict distributed metabolic pathways within defined microbial assemblages e.g., co-cultures or more complex microbial communities in natural and engineered ecosystems.

### Comparative community metabolism

**Figure 4:**
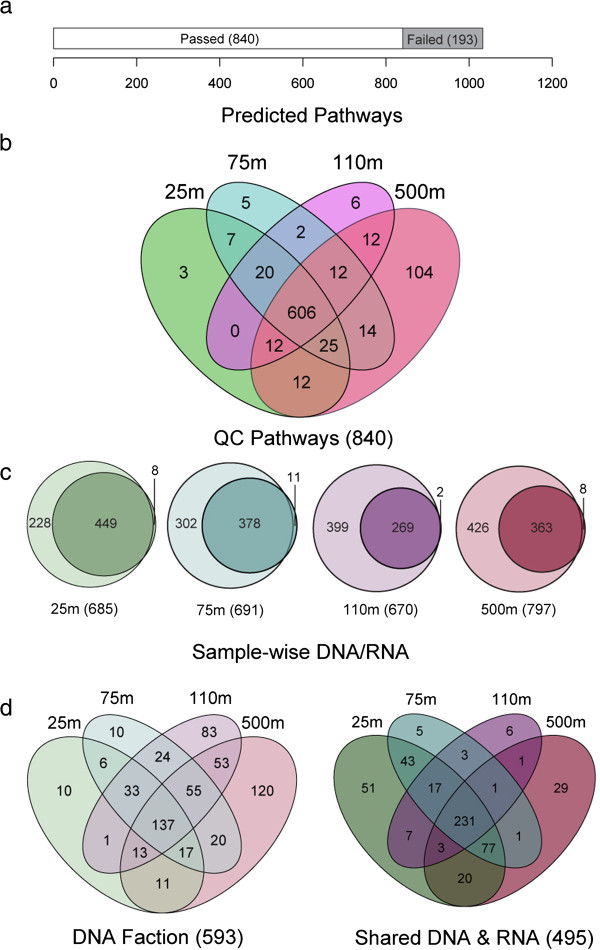
**Analysis of predicted pathways from the Hawaii Ocean Time-series. (a)** A total of 1033 unique pathways were predicted from the HOT samples (Additional file 3), however only 840 unique pathways remained after all pathways in each sample with less than 10 ORFs were removed (Additional file 4). **(b)** After normalizing by total predicted ORFs (Additional files 5 and 6), a 4-way set analysis of these quality controlled (QC) pathways shows that the samples share a large core of common pathways. **(c)** Separating unique pathways within the DNA and RNA of each sample revealed that very few pathways were unique to the RNA fraction of each sample. **(d)** Finally, at set analysis of the unique DNA fraction (light colors), and pathways common to DNA and RNA from each sample (dark colors) found subsets of pathways unique to each fraction (Additional files 7 and 8).

To evaluate Pathway Tools’ performance on complex microbial communities at different information levels we compared and contrasted coupled metagenome (DNA) and metatranscriptome (RNA) datasets from 25, 75, 110 m (sunlit or euphotic) and 500 m (dark) ocean depth intervals from HOT [19]. A total of 1026 unique pathways from approximately 1.2 billion base pairs of environmental sequence information were recovered spanning defined environmental gradients including luminosity, salinity, pressure, and oxygen concentration (Additional file 1: Table S12). Of these pathways, 840 met minimal quality control (QC) standards (Materials and Methods) and were used for subsequent set-difference analysis (Figure 4a).

More than 600 pathways were shared in common between the sunlit and dark ocean based on combined DNA and RNA datasets consistent with a conserved metabolic core (Figure 4b). A total of 14 unique pathways were predicted exclusively in sunlit samples with 20 pathways predicted at the intersection of 25, 75 and 110 m depth intervals (Figure 4b). More than 100 unique pathways were predicted for the 500 m compliment consistent with increased metabolic potential and niche-specialization with increasing depth (Figure 4b). Interestingly, the normalized proportion of genetic potential (DNA) versus expressed metabolic pathways (DNA/RNA) increased linearly between 25, 75 and 110 m depth intervals (0.4, 0.7 and 1.2, respectively) before plateauing at 500 m (1.2) (Figure 4c). It remains to be determined if this trend reflects an asymptote or an inflection point in pathway expression co-varying as a function of metabolic status, environmental conditions or sample coverage and QC.

A total of 30 pathways were identified exclusively in RNA datasets including 11 pathway variants (Figure 4c and Additional file 1: Figure S6). Expressed cholesterol degradation and tetrahydrobiopterin biosynthesis I were common to all depth intervals. Unique expressed photorespiration and glycolate degradation III pathways were recovered at 25 and 75 m, while ammonia oxidation III, methane oxidation to methanol II, and arginine biosynthesis III were unique to 500 m (Additional file 1: Figure S6). More than 590 pathways were identified exclusively in DNA datasets, while 495 were shared in common between DNA and RNA datasets (Figure 4d). With respect to functional classes, unique Degradation, Biosynthesis and Energy-Metabolism pathways increased as a function of depth in DNA datasets (Additional file 1: Figure S7a). Within unique degradation classes a progression from amino acids to aromatic-compounds and secondary metabolites was observed between 25, 75, 110 and 500 m depth intervals. A similar progression was observed for a subset of Biosynthetic classes including polyamines, lipids, and cofactors and for Energy-Metabolism including C1-compounds and fermentation (Additional file 1: Figure S7b).

**Figure 5:**
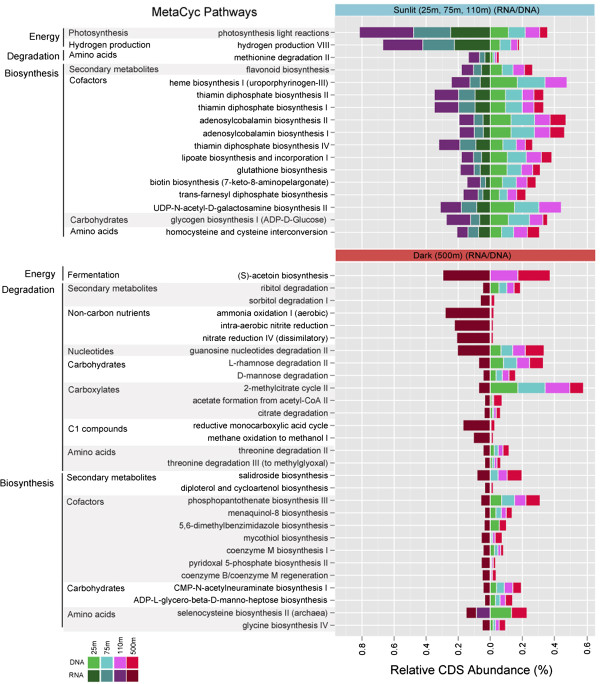
**Comparison of predicted genomic and transcriptomic pathways with unique expression in the ‘sunlit’ and ‘dark’ HOT samples.** Sunlit metabolism was indicative of photosynthesis and aerobic metabolism including photosynthesis light reactions and hydrogen production. Dark metabolism had significantly more degradation pathways.

An evaluation of the 72 most abundant pathways recovered from the combined datasets indicated that 53 were both present and expressed at 25, 75, 110, and 500 m depth intervals. Moreover, several of the most abundant pathways including ammonium transport, Rubisco shunt, NADH to cytochrome electron transfer, pyruvate fermentation, denitrification, Calvin-Benson-Bassham cycle, cysteine biosynthesis I and arginine biosynthesis III exhibited depth-dependent trends in gene expression (Additional file 1: Figure S8). A number of abundant pathways common to 25, 75, 110, and 500 m depth intervals in the DNA datasets were exclusively expressed in sunlit or dark ocean waters (Figure 5). In sunlit waters these included photosynthesis light reactions, hydrogen production VIII, flavonoid biosynthesis, cofactors including heme, vitamin B-complex (thiamin, adenosylcobalamin), and glutathione for oxidative stress (Figure 5). Below the euphotic zone, the 500 m depth interval exclusively expressed pathways for ribitol, rhamnose, guanosine nucleotide, 2-methylcitrate, and threonine degradation as well as pathways for cofactor biosynthesis including phosphopantothenate, menaquinol-8 (vitamin K), and coenzyme M and several carbohydrate and amino acid biosynthetic pathways including CMP-N-acetylneuraminate I, ADP-L-glycero-beta-D-manno-heptose and glycine biosynthesis IV (Figure 5).

Consistent with previous reports, sunlit waters expressed many photosynthesis-related pathways including aerobic electron transfer, hydrogen production, and cofactors including ubiquinol, heme, vitamin B-complex (nicotinate, thiamine, cobalamin, tetrahydrofolate), chlorophyll a, and retinol biosynthesis [19, 20] (Additional file 1: Figures S9 and S10). In addition to photosynthesis, 25 and 75 m depth intervals (upper euphotic) sets included pathways associated with degradation of plant metabolites including phytate, glucuronate, mannitol, chitin, xylose, arabinose, gallate, and quinolate. Other pathways of interest identified in sunlit waters included organophosphate, urea, and aminobutyrate degradation, as well as pathways for conversion of the plant hormone indole-3 acetic acid and mercury detoxification. Below the euphotic zone, the 500 m depth interval expressed unique pathways for intra-aerobic nitrite reduction, dissimilatory nitrate reduction, the reductive monocarboxylic acid cycle, ammonia oxidation, and methane oxidation to methanol I (Additional file 1: Figure S11). Thus, comparative ePGDB analysis using the combined DNA and RNA datasets differentiated between genomic potential and phenotypic expression across defined environmental gradients in the ocean and revealed known and novel patterns of functional specialization with potential implications for nutrient and energy flow within sunlit and dark ocean waters.

### Pathway prediction hazards

While the construction of ePGDBs promotes pathway-centric analysis of environmental sequence information, prediction hazards need to be considered for optimal interpretive power. One common hazard is the ‘multiple mapping problem,’ arising when an enzyme catalyzes conserved or promiscuous reaction steps across multiple pathways or enzyme commission (EC) numbers representing classes with non-specific substrate activity. For example EC 3.2.1.21 represents a non-specific enzyme class for beta-D-glucosides, allowing for spurious prediction of specific carbohydrate degradation pathways. Moreover, PathoLogic has a preference for EC numbers over product descriptions that can further exacerbate false discovery associated with non-specific enzyme classes. Hazards manifesting themselves within pathway variants sharing a number of common or reversible reaction steps have previously been described by Caspi and colleagues in the context of PGDB construction for cellular organisms [28]. For example, the tricarboxylic acid cycle (TCA) cycle has at least eight pathway variants associated with different taxonomic groups and several incomplete or reversible forms that share multiple reactions steps. Pathologic has difficulty differentiating between TCA cycle variants when reversible pathway components are present even when a diagnostic step such as ATP-citrate lyase for the reductive TCA cycle is missing from the input data. A similar problem occurs when a regulatory protein is used to provide evidence that a pathway exists even when catalytic pathway components are missing from the input. Given that we constructed ePGDBs without taxonomic pruning and that PathoLogic uses automated annotations from multiple taxonomic groups when predicting pathways from environmental sequence information, taxon specific pathways such as plant hormone biosynthesis or innate immunity can be predicted even when organisms known to encode such pathways are absent from the dataset. As described in the performance considerations section, WTD can be used to discern differences between the predicted and expected taxonomic range of pathways pointing to potential hazards prior to interpretation. Indeed, the extent to which these predicted pathways reflect previously unrecognized variants or prediction artifacts remains to be determined. Moreover, this hazard has the potential to confound distributed metabolic pathway identification when sequence coverage is low or microbial community composition is extremely uneven. Some examples of these hazards from the HOT analysis are provided in Additional file 1: Table S13.

**Figure 6:**
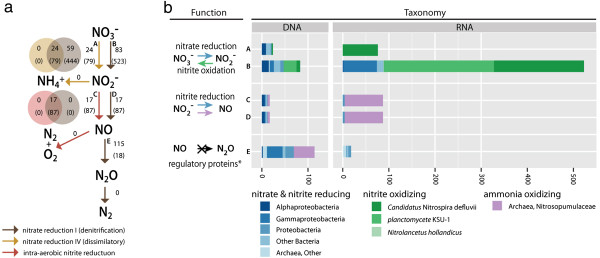
**Taxonomic and functional breakdown of nitrogen cycling pathways.** **(a)** Nitrogen cycling pathways and reactions assigned by PathoLogic. Arrow color indicates pathway, nitrate reduction I (denitrification) (brown), nitrate reduction IV (dissimilatory) (yellow), and intra-aerobic nitrite reduction (red). Grey numbers adjacent to arrows indicated number of reads assigned to the reaction in the DNA and RNA (RNA in parentheses). Overlapping circles indicate the distribution of reads across multiple pathways. **(b)** BLAST-based functional and taxonomic breakdown of reads assigned to reactions in given pathways as indicated by letters A-E. Function was determined by the top RefSeq BLAST hit, reported by the MetaPathways pipeline, and indicated by reaction arrows, with color corresponding to taxa or taxonomic group with known activity: taxa with nitrate and nitrite reducing activity (blue), nitrite oxidizing activity (green), and ammonia oxidizing activity (purple). Grey reactions indicate no reads for enzymatic activity were detected, only regulatory proteins that may be involved in gene expression regulation (*).

The identification of dissimilatory nitrate reduction (denitrification), intra-aerobic nitrite reduction and ammonia oxidation in the combined 500 m HOT DNA and RNA datasets provides a real world example of hazard navigation. Denitrification is a distributed form of energy metabolism resulting in the production of nitrogen gas in oxygen-deficient waters (<20 μM O_2_ per kg) [29, 30]. The first step in denitrification is nitrate reduction to nitrite. In the combined HOT DNA and RNA datasets the predicted pathway variant nitrate reduction IV included a subset of CDS transcripts for ‘nitrate reductase gamma subunit’ (24 in DNA, 79 in RNA) while the predicted pathway variant nitrate reduction I included CDS transcripts for multiple nitrate reductase subunits (Figure 6). While CDS for nitrate reductase subunits originated from a number of different taxa including Alphaproteobacteria, Gammaproteobacteria, Nitrospira and Planctomycetes, 435 out of 523 (83%) predicted nitrate reductase transcripts originated from Nitrospira and Planctomycetes consistent with a role in nitrite oxidation [31–34] (Figure 6). The second step in denitrification is nitrite reduction to nitric oxide. Within the DNA dataset both bacterial and archaeal CDS for nitrite reductase were recovered while transcripts originating from ammonia oxidizing archaea dominated the RNA dataset (Figure 6). Coding sequences/transcripts for downstream pathway components including nitric oxide reductase and nitrous oxide reductase were not detected, although CbbQ/NirQ/NorQ family regulators necessary for inorganic carbon fixation in the Calvin-Benson-Bassham cycle, nitrite and nitric oxide reduction were identified in DNA and RNA datasets [35] (Figure 6). Given that the mean oxygen concentration at 500 m is ~120 μM O_2_ per kg [18, 20], these results are consistent with active water column nitrite and ammonia oxidation processes. Recent studies in the Eastern Tropical South Pacific OMZ observed changes in the frequency distribution of denitrification genes between free-living (0.2-1.6 μm) and particle-associated (>1.6 μm) size fractions, with nitric oxide reductase and nitrous oxide reductase encoding genes enriched on particles [36]. The extent to which denitrification or anammox processes partition between free-living and particle-associated microoganisms in the HOT water column remains to be determined.

## Conclusions

While advances in high throughput sequencing technologies are rapidly giving rise to tens of thousands of environmental datasets, the computational and analytic powers needed to organize, interpret and mobilize these datasets have lagged behind. Conventional BLAST-based annotation methods combined with gene-centric analyses tend to overlook the network properties of microbial communities driving ecological and biogeochemical interactions. We argue that pathway-centric analyses via the MetaPathways pipeline and Pathway Tools provides the scientific user community with an end-to-end solution for comparing ePGDBs constructed from environmental sequence information revealing known and novel network properties. As with any automated analysis, this method is no replacement for manual curation. Indeed, we have highlighted specific instances where taxonomic range, idiosyncratic annotation, multifunctional enzymes, regulatory functions, and reversible enzymatic forms predicted by Pathway Tools result in interpretive hazards that require expert knowledge to resolve.

Continued development efforts are needed to improve on existing features and add new functionality to both the MetaPathways pipeline and Pathway Tools. Specifically, improved import features amenable to categorical metadata e.g., taxonomic origin, location, depth, etc., need to be integrated with Pathway Tools 'groups', a feature that enables users to integrate external data and group pathways and objects within Pathway Tools. The ‘groups’ feature in turn needs to be better integrated into the ‘omics’ viewer allowing for improved pathway navigation and page summaries within the Pathway Tools browser. Tooltip enhancements that summarize the categorical data mentioned above could further enhance the browsing experience. Current ePGDBs are constructed using concatenated CDS sequences and improved viewing features are needed that map coverage and noncoding sequence information onto complete contigs. Finally, the PathoLogic algorithm should be improved to incorporate the described prediction hazards and WTD into its calculations. Specifically, one can imagine tree-based algorithmic improvements to PathoLogic akin to the WTD described here that integrate taxonomic information with enzyme or pathway directionality.

Despite current limitations, ePGDBs provide an interactive and holistic data structure in which to investigate distributed metabolism and differentiate between microbial community metabolic potential and phenotypic expression. Thus, ePGBDs provide a functional blueprint of microbial community metabolism that can be harnessed to engineer microbial consortia with defined emergent properties. These properties can in turn be transferred to industrial strains or modeled using MetaFlux to improve process performance [13]. Although the set-difference and visual inspection methods used to identify distributed metabolic pathways described here do not scale for big datasets, future algorithmic improvements will enable comparisons of reference genomes and metagenomes in large numbers. Indeed, splitting the proverbial “reaction arrows” for each step in a given metabolic pathway into taxonomic bins provides a basis for integer optimization methods that compute “distribution” scores and a baseline for monitoring changes in the reaction network associated with environmental change or even human health status. Looking forward, we envision an open source collection of ePGDBs, called EngCyc analogous to BioCyc [16], which can be queried and compared online revealing the network properties of microbial communities in natural and engineered ecosystems on a truly global scale.

## Methods

### Metabolic pathway analysis

Environmental PGDBs were constructed from public datsets using MetaPathways (http://github.com/hallamlab/MetaPathways/) [14] with default parameter settings: open reading frame (ORF) detection by Prodigal (minimum length 60 amino acids), functional annotation by BLAST (e-value 1e-5, blast-score ratio 0.4) against protein databases KEGG [37], COG [38], MetaCyc [11] (version 16.0), and RefSeq [39] (Downloaded August 2012), and pathway prediction via the PathoLogic algorithm with taxonomic pruning disabled. Predicted pathways and associated annotated CDS sequences were extracted from created ePGDBs using the utility script extract_pathway_table_from_pgdb.pl included with MetaPathways.

### Pathway prediction on simulated data

Simulated sequencing experiments were performed using MetaSim [22] with the parameter settings: Long read: clone size 36000 bp, Gaussian error, mean read length 700 bp, standard deviation 100 bp; Short read: Gaussian error, mean 160 bp, standard deviation 40 bp) against the *E. coli K12 MG1655* complete nucleotide genome (GenBank: NC_000913) at a series of fractional levels (1/32, 1/16, 1/8, 1/4, 1/2, 1/1) of the total combined length of starting component genomes (G_m_). Pathways were predicted using the MetaPathways pipeline, as described above, against each of the resulting sequence sets (Additional file 1: Tables S3 and S4). A classification performance analysis was performed; True positives (TP) were pathways found in both the simulated sample pathways (test set) and the complete gold standard *E. coli* genome. True negatives (TN) were pathways not predicted in the test set or gold standard. False positives (FP) were pathways found in the test set but not in the gold standard. Finally, false negatives (FN) were pathways found in the gold standard but not in the test set. Multiple summary statistics for the resulting confusion tables (Sensitivity (Recall), Specificity, Precision, Accuracy, F-measure, and Matthew’s Correlation Coefficient (MCC)) were calculated. A summary of these performance statistics is provided in the supplement (Additional file 1: Note S1: ‘A Note on Confusion Table Statistics’).

### Simulated metagenomes: Sim1, Sim2

Simulated sequencing experiments of metagenomes Sim1 and Sim2 were generated and analyzed as described above for *E. coli*. To minimize name-mapping problems, we used prokaryotic genomes from the tier-2 BioCyc database collection [21]. The Sim1 metagenome was composed of ten tier-2 BioCyc genomes (Additional file 1: Table S2) in equal copy number, while Sim2 was composed of the *Caulobacter cresentus NA1000* genome in 20-fold excess relative to other genomes (Additional file 1: Figure S1). A classification performance analysis was performed as described above with the set of 646 pathways predicted from the complete tier-2 genomes used to derive Sim1 and Sim2 representing the gold standard (Additional file 1: Tables S5-S8).

### Simulated metagenomes: HOT (25 m)

A 25 m metagenome from the Hawaii ocean time series was sub-sampled with replacement to different fractional levels (1/20, 1/10, 3/20, 1/5, 2/5, 3/5, 4/5, and 1/1) and pathways were predicted as described above. Similarly, a classification performance analysis was performed with the set of 864 pathways predicted from the complete 454 run representing the gold standard (Additional file 1: Tables S7 and S8).

### Taxonomic pruning experiments

The full-G_m_ simulated sequencing samples for Sim1 and Sim2, both short and long read lengths, and the full-G_m_ HOT (25 m) sample, had their pathways predicted with the above method, but with taxonomic pruning enabled using the taxonomic lineage parameter set to “Unclassified sequences”. The number of predicted pathways were tabulated and compared with the pathways previously predicted with taxonomic pruning disabled. As simple set analysis showed that within a sample the pruned pathways were a strict subset of the “no-pruning” ones, and the reduction in pathways was calculated (Additional file 1: Table S9).

### Weighted taxonomic distance

For each predicted pathway in the HOT dataset, a weighted taxonomic distance (WTD) distance was calculated using the WTD algorithm (Additional file 1: Supplementary Note 2). First, the lowest common ancestor algorithm (LCA) was applied to a pathway’s RefSeq CDS sequences. The WTD algorithm calculates a weighted distance *D* between the observed LCA taxonomy *x*_*obs*_ and the pathway’s expected taxonomic range(s) *x*_*exp*_ ∈ *TR*^(*MetaCyc*)^(*p*), where *TR*^(*MetaCyc*)^(*p*) is the set of taxonomic range(s) for a given pathway *p* on the NCBI Taxonomy Database hierarchy.

This WTD algorithm takes as input *p* and *x*_*obs*_, and calculates a weighted taxonomic distance for each *x*_*exp*_ on nodes in the connecting path *P*(*x*_*exp*_, *x*_*obs*_), as


where *e*_*a*,*b*_ is an edge between nodes *a* and *b* in the path and d(a) is the depth of node a. If *x*_*exp*_ descends from the expected taxonomic range *x*_*obs*_, then the WTD is assigned a positive value and WTD for paths descending outside this range are assigned a negative value. After calculating the WTDs for all pairs *x*_*exp*_, *x*_*obs*_, the WTD algorithm first attempts to return the minimum non-negative distance e.g., WTD corresponding to the closest *x*_*exp*_ where *x*_*obs*_ is a descendant of *x*_*exp*_, and returns the maximum negative score e.g., closest to zero if all observed and expected taxonomies diverge. For each dataset, predicted pathways were assigned to a “Disagreement Class” based on the following criteria: (i) pathways with positive WTD were given the “None” class, (ii) pathways with distances greater than the median of negative WTDs were given the “Low” class, (iii) pathways within the 2^nd^ quartile were given the “Medium” class, and (iv) pathways in the lower quartile were given the “High” disagreement class (Additional file 1: Figure S2). The expected taxonomic ranges of each pathway where then collapsed into the higher taxonomic levels: “root”, “cellular organisms”, “prokaryotes”, “archaea”, “bacteria”, “eukaryotes”, “animals”, “fungi”, ”plants”, and “other”, as defined on the NCBI Taxonomy Database hierarchy and pathway frequencies and disagreement classes were summarized for each sample (Additional file 1: Figure S3).

### Distributed metabolic pathway prediction

Four genomes of similar size and complexity from the tier-2 dataset were combined in a pairwise manner: *Aurantimonas manganoxydans SI85-9A* (GenBank: NZ_AAPJ00000000.1), *Bacillus subtilis subtilis 168* (GenBank: AL009126.3), *Caulobacter crescentus NA1000* (GenBank: CP001340.1), and *Helicobacter pylori 26695* (GenBank: AE000511.1), abbreviated by the first character of their proper names, A, B, C, and H, respectively. The six pair-wise and four original genomes were analyzed as described above for *E. coli* (Additional file 1: Table S10). Pathways predicted in the combined PGDBs were considered candidates for distributed metabolism if they were absent from PGDBs for individual genomes (i.e., found in A and B combined, but not in either A or B individually) (Additional file 1: Table S11 and Additional file 2). Candidate pathways were manually inspected and deemed ‘plausible’ if there was sufficient coverage, i.e., 75% of reactions in a pathway had associated CDS sequences from both taxa (Additional file 1: Figure S4).

Similarly, the *Candidatus Moranella endobia* and *Candidatus Tremblaya princeps* genomes (GenBank: NC-015735 and NC-015736) were downloaded from NCBI and analyzed as described above for *E. coli*. Resulting PGDBs for individual and combined genomes were manually inspected for amino acid biosynthetic pathways described in McCutcheon and Dohlen [17] (Additional file 1: Figure S5).

### Hawaii ocean time-series

Unassembled metagenomic and transcriptomic pyrosequences from the Hawaii Ocean Time-series (10 m, 75 m, 110 m, and 500 m) were obtained from the NCBI Sequence Read Archive (SRA Accession: SRX007372, SRX007369, SRX007370, SRX007371, SRX016893, SRX016897, SRX156384, SRX156385) and run through the MetaPathways pipeline using default settings (Additional file 3). To avoid spurious predictions, only pathways with more than ten mapped CDS sequences in an individual sample were used in downstream analysis. The pathways with nine or fewer mapped CDS sequences represent the lower quartile of pathway annotations (Figure 4a, Additional file 4). Pathway CDS counts for each sample were normalized to the total number of unannotated ORFs in each dataset. Count data was then converted to percentages providing relative ORF abundance for each pathway (Additional file 5), along with their weighted taxonomic distances and sample-wise disagreement classes (Additional file 6). Relative CDS abundance of the top-40 pathways from DNA and RNA datasets were compared (Additional file 1: Figure S8). In addition, pathways predicted in the DNA and RNA datasets were compared at each depth interval to provide sample-wise fractions for each depth e.g., DNA-only, DNA-RNA, and RNA-only (Figure 4c). Given the small number of pathways in the RNA-only sets no set-difference analysis was needed (Additional file 1: Figure S6). The DNA-only sets were declined and tabulated at various levels of the MetaCyc pathway hierarchy (Additional file 1: Figure S7). A final four-way set analysis was performed on the DNA-only and DNA-RNA pathways at each depth (Figure 4d, Additional files 7 and 8). DNA-RNA set-difference subsets with more than 5 predicted pathways were compared in detail (Additional file 1: Figures S9-S14). All data transformations, set operations, and comparisons were performed in the R statistical environment (http://www.r-project.org), and visualized using the ggplot graphical package (http://ggplot2.org) and d3.js graphical library (http://d3js.org/).

### Availability of supporting data

The ten full-length genomes used to create simulated metagenomes can be downloaded from GenBank under accession numbers AE008687-AE008690, NZ_AAPJ00000000.1, AL009126.3, AE005673, CP001340.1, AE000511.1, AE000516, AL123456, NC_007604.1, AE003852, and AE003853.

The symbiotic *Candidatus Moranella endobia* and *Candidatus Tremblaya princeps* genomes can be downloaded from GenBank under accession numbers NC-015735 and NC-015736). The Hawaii Ocean Time series datasets can be downloaded from the NCBI Sequence Read Archive under accession numbers SRX007372, SRX007369, SRX007370, SRX007371, SRX016893, SRX016897, SRX156384, SRX156385.

## Electronic supplementary material

Additional file 1: **Supplementary notes, figures, and tables.** (PDF 12 MB)

Additional file 2: **Summary of candidate pathways that are potentially distributed by set-difference analysis.** (XLS 30 KB)

Additional file 3: **Summary table of 1033 pre-QC predicted pathways and CDS counts for the Hawaii Ocean Time-series samples.** (XLS 217 KB)

Additional file 4: **Summary table of the 840 post-QC predicted pathways and CDS counts for the Hawaii Ocean Time-series samples.** (XLS 183 KB)

Additional file 5: **Summary table of the 840 post-QC predicted pathways and normalized CDS counts for the Hawaii Ocean Time-series samples with taxonomic disagreement class highlighted.** (XLS 304 KB)

Additional file 6: **Summary table of the 840 post-QC predicted pathways and normalized CDS counts for the Hawaii Ocean Time-series samples with observed LCA taxonomies, expected taxonomic ranges, calculated weighted taxonomic distance, and taxonomic disagreement class.** (XLS 806 KB)

Additional file 7: **Summary table of normalized CDS counts for the 593 DNA fraction pathways of samples from the Hawaii Ocean Time-series.** (XLS 150 KB)

Additional file 8: **Summary table of normalized CDS counts for the 495 pathways common to DNA and RNA samples from the Hawaii Ocean Time-series.** (XLS 168 KB)
