# Supplementary material for: Metabolic pathways for the whole community
Source: BMC Genomics. 2014 Jul 22;15(1):619. doi: 10.1186/1471-2164-15-619 (PMC4137073; doi:10.1186/1471-2164-15-619)
Supplement: Supplementary file 1 — Additional file 1: Supplementary notes, figures, and tables. (PDF 12 MB) [file 12864_2014_6353_MOESM1_ESM.pdf]

Additional File 1: Supplementary Material for Metabolic pathways for the whole community.

Table S1. Overview of the *E. coli K12* genome used for simulated sequencing experiments.

Taxa	GenBank	Size (bp)	Genes
Escherichia coli str. K-12 substr. MG1655	NC_000913	4,639,675	4,288

Table S2. Overview of the tier-2 BioCyc genomes used for simulated sequencing experiments.

Taxa	GenBank	Size (bp)	Genes
Agrobacterium tumefaciens C58	AE008687-AE008690	5,674,064	5,469
Aurantimonas manganoxydans SI85-9A1	NZ_AAPJ00000000.1	4,285,343	3,665
Bacillus subtilis subtilis 168	AL009126.3	4,215,606	4,428
Caulobacter crescentus CB15	AE005673	4,016,947	3,819
Caulobacter crescentus NA1000	CP001340.1	4,042,929	3,968
Helicobacter pylori 26695	AE000511.1	1,667,867	1,609
Mycobacterium tuberculosis CDC1551	AE000516	4,403,836	4,235
Mycobacterium tuberculosis H37Rv	AL123456	4,411,529	3,916
Synechococcus elongatus PCC 7942	NC_007604.1	2,695,903	2,664
Vibrio cholerae O1 biovar El Tor str. N16961	AE003852, AE003853	4,033,464	3,952
**Total**		39,447,488	37,725
**Average**		3,944,749	3,773

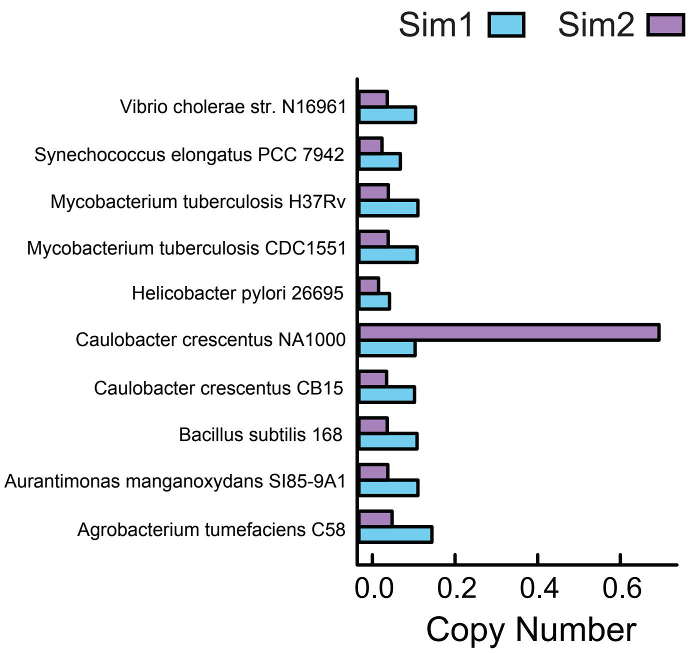

Figure S1. Copy number distributions for the simulated metagenomes Sim1 and Sim2. Sim1 (blue) has the ten BioCyc taxa in approximately equal proportion. Sim2 (purple) has the genome copy number of *Caulobacter crescentus NA1000* in approximately twenty times abundance. Taxa used were selected with approximately equal genome size and gene content.

Table S3. Overview of Long-read simulated sequencing experiments for *E. coli K12,* Sim1, and Sim2 taxonomic distributions.

Distribution	G_m	Size (bp)	Reads	ORFs	Annotated CDS	Predicted Pathways	Recovered (%)
E. coli K12	0.03	143,339	180	243	150	71	0.20
E. coli K12	0.06	285,694	360	494	309	85	0.24
E. coli K12	0.12	575,821	720	973	604	154	0.44
E. coli K12	0.25	1,151,303	1,438	1,992	1,207	206	0.58
E. coli K12	0.50	2,305,899	2,876	4,030	2,401	271	0.77
E. coli K12	1.0	4,594,877	5,750	7,996	4,859	352	0.99
Sim1	0.03	1,249,104	1,564	2,978	771	208	0.32
Sim1	0.06	2,501,345	3,126	5,857	1,487	268	0.41
Sim1	0.12	5,003,783	6,250	11,769	3,120	392	0.61
Sim1	0.25	10,014,496	12,500	23,422	6,107	475	0.74
Sim1	0.5	19,991,551	25,000	47,304	12,139	542	0.84
Sim1	1.0	40,016,291	50,000	94,438	24,388	604	0.93
Sim2	0.03	1,245,781	1,562	2,946	760	175	0.27
Sim2	0.06	2,496,313	3,126	5,880	1538	247	0.38
Sim2	0.12	4,987,646	6,250	11,756	3154	339	0.52
Sim2	0.25	9,994,331	12,500	23,852	6330	425	0.66
Sim2	0.50	19,993,717	25,000	47,350	12,676	471	0.73
Sim2	1.0	40,006,531	50,000	94,366	25,139	550	0.85

Table S4. Overview of Short-read simulated sequencing experiments for *E. coli K12,* Sim1, Sim2, and HOT (25m) taxonomic distributions.

Distribution	G_m	Size (bp)	Reads	ORFs	Annotated CDS	Predicted Pathways	Pathways Recovered (%)
E. coli K12	0.03	139,983	540	179	37	9	0.03
E. coli K12	0.06	292,642	1,125	356	68	27	0.08
E. coli K12	0.12	584,031	2,250	742	128	51	0.14
E. coli K12	0.25	1,168,390	4,500	1,445	269	96	0.27
E. coli K12	0.50	2,340,834	9,000	2,878	516	121	0.34
E. coli K12	1.0	4,676,245	18,000	5,884	1,013	181	0.51
Sim1	0.03	1,283,742	4,738	4,151	2,261	108	0.17
Sim1	0.06	2,570,031	9,476	8,266	4,576	169	0.26
Sim1	0.12	5,140,469	18,975	16,549	9,132	239	0.37
Sim1	0.25	10,271,637	37,904	33,164	18,270	316	0.49
Sim1	0.50	20,540,345	75,808	66,260	36,443	431	0.67
Sim1	1.0	41,097,945	151,616	132,577	72,937	499	0.77
Sim2	0.03	1,282,621	4,738	4,337	2,666	113	0.17
Sim2	0.06	2,567,379	9,476	8,657	5,193	171	0.26
Sim2	0.12	5,133,838	18,952	17,313	10,496	237	0.37
Sim2	0.25	10,264,228	37,904	34,624	21,301	334	0.52
Sim2	0.50	20,545,013	75,808	69,256	41,901	392	0.61
Sim2	1.0	41,074,096	151,616	138,593	83,929	502	0.78
HOT (25m)	0.05	8,012,746	31,178	6,668	5,978	336	0.42
HOT (25m)	0.10	16,025,492	62,356	13,478	12,087	398	0.50
HOT (25m)	0.15	24,038,238	93,534	20,054	17,953	438	0.55
HOT (25m)	0.20	32,050,984	124,712	26,836	23,972	462	0.58
HOT (25m)	0.40	64,101,968	249,424	53,300	47,617	526	0.66
HOT (25m)	0.60	96,152,695	374,135	80,080	71,599	555	0.70
HOT (25m)	0.80	128,203,679	498,847	106,985	95,766	585	0.73
HOT (25m)	1.0	160,254,663	623,559	133,836	119,867	593	0.74

Note S1: Confusion Table Statistics

In machine learning a *confusion table* (*contingency table*) is a method to assess the performance of a supervised classifier. Rows of the table represent class predictions, while columns represent the actual class. Given a predicted class and the known class, there are four possible outcomes for the prediction:

Correct Responses

True Positives (TP) - The classifier correctly identified the class as present.
True Negatives (TN) - The classifier correctly identified the class as absent.

Incorrect Responses

False Positives (FP) (Type 1 Error) - The classifier incorrectly predicted the class present when absent.
False Negative (FN) (Type 2 Error) - The classifier incorrectly predicted the class absent when present.

		Actual Class		
		Positive	Negative	
Prediction	Positive	True Positives (TP)	False Positives (FP)	Precision<br>TP / (TP + FP)
	Negative	False Negatives (FN)	True Negatives (TN)	Negative Predictive Value<br>TN / (FN + TN)
		Sensitivity<br>TP / (TP + FN)	Specificity<br>TN / (FP + TN)	

Summary Statistics

Since classifiers can have very different performance characteristics it is often important to consider different statistics of the confusion table. In most situations, there is often a trade off between the two types of errors that a classifier can make.

Sensitivity (Recall) – Represents the ability of the classifier to find positive results. Given that a class is actually in the sample, what is the probability that it is found? High values represent a low number of false negatives (Type-II errors).

Sensitivity = (# correctly predicted present) / (# actually present)

Sensitivity = TP / (TP + FN)

Specificity - The ability of the test to find negative results. What is the probability of correctly rejecting a class. High values represent a low number of false positives (Type-I errors).

Specificity = (# predicted absent) / (# actually absent)

Specificity = TN / (FP + TN)

Precision - Given a positive prediction, what is the probability that it is correct? High values represent a low number of false positives (Type-I errors).

Precision = (# correctly predicted present) / (# predicted present)

Precision = TP / (TP + FP)

Negative Predictive Value (NPV) - Given a negative prediction, what is the probability of actually being correct? High values represent low false negatives (Type-2 errors).

NPV = (# correctly predicted absent) / (# predicted absent)

NPV = TN / (FN + TN)

Ideally one will investigate the confusion table directly, however, because it is onerous to compare many tables, a number of statistics have been developed to summarize the performance described in a table.

Accuracy is the most intuitive, but can be misleading if the distribution of positive and negative results are not of similar magnitude. It asks, of all the decisions that the classifier made, how many were correct?

Accuracy = (Number of correct predictions) / (Total Predictions)

Accuracy = (TP + TN) / (TP + TN + FP + FN)

E.g. Which classifier is the best: A, B, C, or D?

	TP	*TN*	*FP*	*FN*	*Accuracy (%)*
A	25	75	25	75	50
B	0	150	0	50	75
C	50	0	150	0	25
D	30	100	50	20	65

F-measure is the harmonic mean between precision and sensitivity. Therefore, it represents the number of correctly predicted values scaled between false-positive and false-negative errors. However, it does not take into account the number of true-negative responses, which can be important depending on the application.

$$\text{F-measure} = 2 \times \frac{\text{precision} \times \text{sensitivity}}{\text{precision} + \text{sensitivity}}$$

Matthew's Correlation Coefficient (MCC) is a comprehensive measure that controls for the population differences between total positive and negatives in a test or training sample. Essentially it is a correlation coefficient between observed and predicted responses where +1 is perfect prediction, -1 is total disagreement, and 0 is no better than randomly guessing (i.e. no correlation).

$$MCC = \frac{(TP)(TN)-(FP)(FN)}{\sqrt{(TP+FP)(TP+FN)(TN+FP)(TN+FN)}}$$

Matthews Correlation is generally accepted to be the best overall summary statistic of a confusion table that is robust to unequal class sizes, as well as taking into account both Type-1 and Type-2 errors.

Table S5. Overview of pathway prediction performance for simulated Long-read metagenomes of *E. coli K12,* Sim1, and Sim2 at progressively larger genomic sequence coverage.

Distribution	G_m	Precision	Sensitivity	Specificity	Accuracy	F-measure	Matthews
E. coli K12	0.03	0.93	0.18	1.00	0.83	0.31	0.83
E. coli K12	0.06	0.70	0.18	0.98	0.81	0.28	0.56
E. coli K12	0.12	0.76	0.32	0.97	0.84	0.45	0.64
E. coli K12	0.25	0.85	0.50	0.98	0.88	0.63	0.77
E. coli K12	0.50	0.81	0.62	0.96	0.89	0.71	0.74
E. coli K12	1.0	0.84	0.85	0.96	0.93	0.84	0.80
Sim1	0.03	0.96	0.31	0.99	0.73	0.47	0.79
Sim1	0.06	0.91	0.38	0.98	0.75	0.53	0.73
Sim1	0.12	0.94	0.57	0.98	0.82	0.71	0.81
Sim1	0.25	0.94	0.69	0.97	0.86	0.80	0.83
Sim1	0.50	0.95	0.80	0.98	0.91	0.87	0.88
Sim1	1.0	0.95	0.89	0.97	0.94	0.92	0.91
Sim2	0.03	0.93	0.25	0.99	0.70	0.40	0.74
Sim2	0.06	0.95	0.36	0.99	0.75	0.53	0.78
Sim2	0.12	0.93	0.49	0.98	0.79	0.64	0.78
Sim2	0.25	0.95	0.62	0.98	0.84	0.75	0.83
Sim2	0.50	0.97	0.70	0.98	0.88	0.81	0.87
Sim2	1.0	0.95	0.81	0.97	0.91	0.87	0.87

Table S6. Confusion tables of pathway prediction using simulated Long-read sequencing upon the *E. coli K12* genome, Sim1, and Sim2 at progressively larger genomic sequence coverage.

Distribution	G_m	TP	TN	FP	FN	P	N
E. coli K12	0.03	71	1453	5	317	76	1770
E. coli K12	0.06	69	1428	30	319	99	1747
E. coli K12	0.12	124	1418	40	264	164	1682
E. coli K12	0.25	195	1423	35	193	230	1616
E. coli K12	0.50	242	1402	56	146	298	1548
E. coli K12	1.0	328	1397	61	60	389	1457
Sim1	0.03	200	1023	8	446	208	1469
Sim1	0.06	244	1007	24	402	268	1409
Sim1	0.12	368	1007	24	278	392	1285
Sim1	0.25	446	1002	29	200	475	1202
Sim1	0.50	517	1006	25	129	542	1135
Sim1	1.0	576	1003	28	70	604	1073
Sim2	0.03	163	1019	12	483	175	1502
Sim2	0.06	235	1019	12	411	247	1430
Sim2	0.12	316	1008	23	330	339	1338
Sim2	0.25	403	1009	22	243	425	1252
Sim2	0.50	455	1015	16	191	471	1206
Sim2	1.0	521	1002	29	125	550	1127

Table S7. Overview of pathway prediction performance for simulated Short-read sequencing experiments of the *E. coli K12*, Sim1, Sim2, and HOT 25m metagenome at progressively larger genomic sequence coverage.

Distribution	G_m	Precision	Sensitivity	Specificity	Accuracy	F-measure	Matthews
E. coli K12	0.03	1.00	0.03	1.00	0.79	0.05	0.89
E. coli K12	0.06	0.78	0.06	1.00	0.80	0.11	0.64
E. coli K12	0.12	0.88	0.13	1.00	0.81	0.22	0.77
E. coli K12	0.25	0.76	0.21	0.98	0.82	0.32	0.64
E. coli K12	0.50	0.84	0.29	0.99	0.84	0.43	0.74
E. coli K12	1.0	0.85	0.43	0.98	0.86	0.57	0.76
Sim1	0.03	0.91	0.15	0.99	0.67	0.26	0.69
Sim1	0.06	0.86	0.22	0.98	0.69	0.36	0.64
Sim1	0.12	0.88	0.33	0.97	0.72	0.48	0.69
Sim1	0.25	0.86	0.42	0.96	0.75	0.57	0.67
Sim1	0.5	0.87	0.58	0.94	0.80	0.69	0.71
Sim1	1.0	0.85	0.65	0.93	0.82	0.74	0.70
Sim2	0.03	0.88	0.15	0.99	0.67	0.26	0.65
Sim2	0.06	0.87	0.23	0.98	0.69	0.36	0.65
Sim2	0.12	0.87	0.32	0.97	0.72	0.47	0.67
Sim2	0.25	0.88	0.46	0.96	0.77	0.60	0.71
Sim2	0.5	0.89	0.54	0.96	0.79	0.67	0.73
Sim2	1.0	0.86	0.67	0.93	0.83	0.76	0.73
HOT (25m)	0.05	0.96	0.41	0.99	0.71	0.57	0.75
HOT (25m)	0.10	0.97	0.48	0.99	0.75	0.65	0.78
HOT (25m)	0.15	0.96	0.53	0.98	0.77	0.68	0.78
HOT (25m)	0.20	0.96	0.56	0.98	0.78	0.70	0.78
HOT (25m)	0.40	0.95	0.63	0.97	0.81	0.76	0.80
HOT (25m)	0.60	0.95	0.66	0.97	0.82	0.78	0.80
HOT (25m)	0.80	0.94	0.69	0.96	0.83	0.80	0.80
HOT (25m)	1.0	0.95	0.70	0.96	0.84	0.81	0.81

Table S8. Confusion tables of pathway prediction for simulated Short-read sequencing experiments of the *E. coli K12*, Sim1, Sim2, and the HOT 25 m metagenome at progressively larger genomic sequence coverage.

Distribution	G_m	TP	TN	FP	FN	P	N
E. coli K12	0.03	9	1323	0	345	9	1668
E. coli K12	0.06	21	1317	6	333	27	1650
E. coli K12	0.12	45	1317	6	309	51	1626
E. coli K12	0.25	73	1300	23	281	96	1581
E. coli K12	0.50	102	1304	19	252	121	1556
E. coli K12	1.0	153	1295	28	201	181	1496
Sim1	0.03	98	1021	10	548	108	1569
Sim1	0.06	145	1007	24	501	169	1508
Sim1	0.12	211	1003	28	435	239	1438
Sim1	0.25	272	987	44	374	316	1361
Sim1	0.5	373	973	58	273	431	1246
Sim1	1.0	423	955	76	223	499	1178
Sim2	0.03	99	1017	14	547	113	1564
Sim2	0.06	148	1008	23	498	171	1506
Sim2	0.12	206	1000	31	440	237	1440
Sim2	0.25	295	992	39	351	334	1343
Sim2	0.5	347	986	45	299	392	1285
Sim2	1.0	434	963	68	212	502	1175
HOT (25m)	0.05	323	868	13	473	336	1341
HOT (25m)	0.10	386	869	12	410	398	1279
HOT (25m)	0.15	420	863	18	376	438	1239
HOT (25m)	0.20	443	862	19	353	462	1215
HOT (25m)	0.40	501	856	25	295	526	1151
HOT (25m)	0.60	526	852	29	270	555	1122
HOT (25m)	0.80	552	848	33	244	585	1092
HOT (25m)	1.0	561	849	32	235	593	1084

Table S9. Results of taxonomic pruning pathway recovery experiments for simulated metagenomes Sim1 and Sim2 and the HOT 25 m metagenome using the 'Unclassified sequences' taxonomic parameter.

Distribution	Read Length	Pruning	No Pruning	Reduction (%)
Sim1	Long	260	604	56.95
Sim1	Short	194	499	61.12
Sim2	Long	222	550	59.64
Sim2	Short	184	502	63.35
HOT (25m)	N/A	425	593	28.33

Supplementary Note 2. The Weighted Taxonomic Distance (WTD) algorithm

Introduction

The MetaCyc database contains a variety of genes and pathways affiliated with different taxonomic ranges. Thus pathway prediction using the PathoLogic algorithm typically involves a taxonomic pruning component based on the curated "expected taxonomic-range" for each pathway, penalizing the prediction of pathways outside of this specified range. While pruning can reduce false discovery when conducting metabolic reconstruction on individual genomes, environmental sequence information encompasses diverse donor genomes representing numerous taxonomic groups spanning multiple domains of life. A more appropriate distance measure would incorporate the expected taxonomic range(s) provided by MetaCyc and the observed taxa associated with the RefSeq annotated CDS sequences assigned to each pathway. This would assist with the interpretation of predicted pathways by providing a contextual measure of taxonomic agreement between observed and expected taxonomic range for each pathway. Here we describe the derivation, applicability, and use of a weighted taxonomic distance (WTD) algorithm for making such a comparison, incorporating the NCBI Taxonomy Database and the Lowest Common Ancestor (LCA) algorithm [23].

NCBI Taxonomy Database

Organismal taxonomy is a powerful organizing principle based on central tenants of evolution: "inheritance, homology by common descent, and the conservation of sequence and structure". The NCBI Taxonomy provides a curated database consisting of organismal sequences submitted to Genbank. As of April 2014, more than 300,000 taxonomic records were contained within the NCBI Taxonomy Database. Despite this large size, only about 50% of all birds and mammals are represented, and many taxonomic groups have no or limited representation. Manually curated and hierarchically structured, the NCBI Taxonomy Database classification scheme is based on the unison of morphological and molecular taxonomy methods that approximate the evolutionary relationships between extant life forms.

Due to its hierarchical structure, we can view the database as a tree where each node represents an individual taxon. For convenience we will denote this tree T_{NCBI} and henceforth refer to it as the NCBI Tree. The root node represents the root of the tree, which by definition, has every taxon node contained within its sub-tree. A path from the root to any taxon is called a *lineage* consisting of all taxa along that path. Each node in the database can have synonyms that represent the same taxon, but a numerical identifier called a *taxid* uniquely represents each node.

Let us first denote NCBI Tree as $T_{NCBI} = (V, E)$ with vertex and edge sets V and E corresponding to the taxa nodes and their relationships, respectively. Since T_{NCBI} is a tree, it has no cycles, meaning the shortest path between any two nodes $a, b \in V$ is *unique*. We will also assume that the set of edges E is *undirected*. For the purpose of providing a measure of taxonomic distance, we would like a distance, over the NCBI Tree, which captures the notion of taxonomic separation and reflects certain observations about evolution. Below we describe these observations and the expected behavior of a hypothetical distance measure D_{hyp} that respects these observations. We will use an example illustrative graph as an intuitive aid.

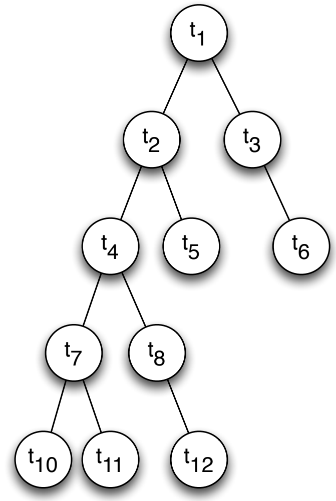

Observation 1: Taxonomic distance represented by edge weight generally decreases with the depth of the tree. Splits near the root of the tree represent major evolutionary distances e.g., Domains (Bacteria, Archaea, and Eukaryotes), while splits closer to the tree tips represent more recently diverged taxa. Intuitively this means that edge weights should decrease with respect to their depth in the tree. For example, in the graph above the following inequalities should hold:

$$D_{hyp}(t_4, t_7) < D_{hyp}(t_2, t_4) < D_{hyp}(t_1, t_2)$$

Observation 2: A split between two taxa in the NCBI tree separates two taxa from a common ancestor. More specifically, if taxon A is not in the lineage of taxon B, the two taxa A and B diverge into two separate lineages. Here, the divergent distance from taxon A to taxon B should be larger than the distance from taxon A to taxon C, where C is a descendant of A i.e., A is in the lineage of C. A distance D_{hyp} should respect this divergence; for example, the following should hold in the illustrative graph above:

$$D_{hyp}(t_4, t_{10}) < D_{hyp}(t_4, t_5)$$

Observation 3: Divergence distance increases with tree depth. Splits nearer the tree tips represent a smaller taxonomic distance than those closer to the root i.e., the taxonomic significance of speciation events is inversely proportional to their depth in the tree. Thus, we expect the following example inequality to hold in the illustrative graph above:

$$D_{hyp}(t_{10}, t_5) < D_{hyp}(t_2, t_3)$$

Weighted Taxonomic Distance Formulation

We will now derive a weighted taxonomic distance D between two nodes on the NCBI Tree that respects the above three observations. First, let us define the depth of a node a

as the number of edges in its shortest-path to the root of the tree, and denote this $d(a)$. Note that the depth of the root node a_{root} is zero, $d(a_{root}) = 0$. Consider any set of links or edges between nodes k and l $e_{k,l} \in E$ and assume without loss of generality, $d(k) < d(l)$. We will define the *lineage* of a taxon a as the set of all taxa along the shortest path from a to a_{root} inclusive, and denote this $L(a)$. We will say that a taxon b is *in the lineage of* a if $b \in L(a)$. Note that this has the evolutionary interpretation that b is an ancestor of a (or is a itself).

Definition: Consider any edge $e_{k,l}$ in the NCBI Tree T_{NCBI} between the adjacent nodes k and l. Without loss of generality, since the edges are undirected, assume that $d(k) < d(l)$, and we define the weight of the edge $e_{k,l}$ as $c(e_{k,l}) = \frac{1}{2^{d(k)}}$. Next, we define the distance between two taxa $a, b \in V$ as

$$D(a,b) \equiv \sum_{e_{x,y} \in E_{P(a,b)}} c(e_{x,y}) = \sum_{e_{x,y} \in E_{P(a,b)}} \frac{1}{2^{d(x)}},$$

where $P(a,b)$ is a subset of edges in E along the shortest path between a and b.

We would like to emphasize that the above distance measure conforms to our three observations. Although the function $c(e_{x,y})$ could be any monotonically decreasing function with the depth of the tree, in defining $D(a,b)$, the integral negative power of two has the following algebraic property:

$$\sum_{j=i+1}^{n} \frac{1}{2^j} < \frac{1}{2^i} \equiv \sum_{j=i+1}^{\infty} \frac{1}{2^j}$$

for any positive integer n and non-negative integer i. How this property affects distances in a tree is best illustrated by a quick illustrative example below.

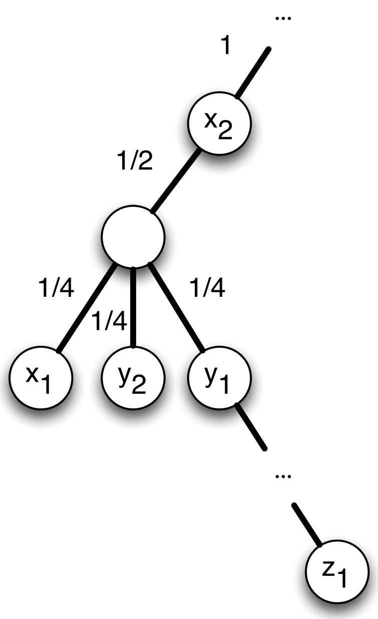

Observe that the distance $D(x_1, y_1) = \frac{1}{4} + \frac{1}{4} = \frac{1}{2}$ is strictly smaller than distance $D(x_2, y_2) = \frac{1}{4} + \frac{1}{2} = \frac{3}{4}$. However, what is more interesting is that $D(x_1, z_1)$ is strictly smaller than $D(x_2, y_2)$ for all possible child nodes of y_1, illustrated as z_1 above.

$$D(x_1, z_1) < D(x_2, y_2)$$

$$\frac{1}{4} + \left(\frac{1}{4} + \frac{1}{8} + \cdots\right) < \frac{1}{2} + \frac{1}{4}$$

Now the convenience of our choice of $c(e_{x,y})$ with respect to the three observations can be shown more explicitly:

Observation 1. Consider two pairs of taxa (a_1, a_2) and (b_1, b_2), such that: (i) a_1 is *in the lineage of* a_2 and (ii) b_1 is *in the lineage of* b_2, and (iii) the number of edges between $a_1$and a_2, and b_1 and b_2 are equal, say *k*; then if $d(a_1) \leq d(b_1)$ implies $D(a_1, a_2) \geq D(b_1, b_2)$, and similarity $d(a_1) > d(b_1)$ implies $D(a_1, a_2) < D(b_1, b_2)$. To show this, without loss of generality suppose $d(a_1) \leq d(b_1)$, then

$$D(a_1, a_2) \equiv \sum_{i=0}^{k-1} \frac{1}{2^{d(a_1)+i}} \geq \sum_{i=0}^{k-1} \frac{1}{2^{d(b_1)+i}} \equiv D(b_1, b_2)$$

Observation 2. Consider the following three taxa t_1, t_2, and t_3 such that: (i) t_1 is *in the lineage of* t_2 and (ii) t_1 and t_3 have *diverged*, i.e., $t_1$is not *in the lineage of* t_3. Then it follows that $D(t_1, t_2) < D(t_1, t_3)$.

To see this, note that since t_1 is in the lineage of t_2 then $d(t_1) \leq d(t_2)$, but since t_1 and t_3 have diverged there exists a common parent p where the divergence occurred, implying $d(p) < d(t_1)$. Thus the following holds:

$$D(t_1, t_3) > \frac{1}{2^{d(p)}} > \sum_{i=0}^{\infty} \frac{1}{2^{d(t_1)+i}} \geq D(t_1, t_2)$$

Observation 3. This can be shown, in a similar manner to Observation 1, by considering two pairs of divergent taxa (a_1, a_2) and (b_1, b_2) where the common parents of (a_1, a_2) and (b_1, b_2) are $p_{(a_1,a_2)}$ and $p_{(b_1,b_2)}$, respectively. If $d\left(p_{(a_1,a_2)}\right) < d\left(p_{(b_1,b_2)}\right)$, then $D(a_1, a_2) > D(b_1, b_2)$, if $d\left(p_{(a_1,a_2)}\right) > d\left(p_{(b_1,b_2)}\right)$, then $D(a_1, a_2) < D(b_1, b_2)$. Then without loss of generality, suppose $d\left(p_{(a_1,a_2)}\right) < d(p_{(b_1,b_2)})$. Now,

$$D(a_1, a_2) = D\left(p_{(a_1,a_2)}, a_1\right) + D(p_{(a_1,a_2)}, a_2)$$

$$\geq \frac{1}{2^{d(p_{(a_1,a_2)})}} + \frac{1}{2^{d(p_{(a_1,a_2)})}} \geq \sum_{i=0}^{\infty} \frac{1}{2^{d(p_{(b_1,b_2)})+i}} + \sum_{i=0}^{\infty} \frac{1}{2^{d(p_{(b_1,b_2)})+i}}$$

$$> D\left(p_{(b_1,b_2)}, b_1\right) + D\left(p_{(b_1,b_2)}, b_2\right) = D(b_1, b_2)$$

where the first inequality in the above comes from considering only the first edge in the walk from $p_{(a_1,a_2)}$ to a_1 and $p_{(a_1,a_2)}$ to a_2.

Lowest Common Ancestor (LCA) Algorithm

The LCA algorithm traverses the NCBI Tree to return the lowest common ancestor of a set of taxa [23]. Two lineages are considered highly evolutionarily diverged if their LCA is near the root of the hierarchy i.e., a high level rank such as phylu), while lineages whose LCA is near the leaves of the hierarchy i.e., a low level rank like genus are less divergent. In MEGAN this is used to place annotated sequences on the NCBI Tree by applying the LCA algorithm to the set of taxa found in its RefSeq BLAST hits. The WTD algorithm calculates LCA using the set of all taxa found in the CDS annotations associated with enzyme reactions of a pathway. Lets define LCA in more precise terms. Let $T = \{t_1, t_2, \ldots, t_n\}$ be a set of taxa in the NCBI Tree T_{NCBI} and let P be the set of taxa, such that $\forall\, p \in P$, p is a parent or indirect parent of all taxa in T. Note that $P \neq \emptyset$ since $a_{root} \in P$. Now, the *lowest common ancestor* $LCA(T)$ is the taxon $p \in P$ which has the lowest depth in the tree.

The Weighted Taxonomic Distance Algorithm

Now that we have described the weighted taxonomic distance $D(\cdot,\cdot)$ between two nodes on the NCBI Tree, and have described the LCA algorithm we can discuss the specifics of how the WTD algorithm uses both the distance and LCA to reconcile the two different sources of taxonomic annotations of predicted MetaCyc pathways. Recall that there are two sources of taxonomies associated with a pathway: (i) the observed taxonomic annotations for CDS sequences found for pathway reaction enzymes and (ii) the expected

taxonomic range(s) from MetaCyc, a set of taxa curated for many MetaCyc pathways. For a given pathway, the set of CDS annotations are input in the LCA algorithm, returning their observed LCA taxon. Next, the distance between observed LCA taxon is compared with each member of the pathway's expected taxonomic range(s), returning the distance with the smallest magnitude, with a preference for positive distances (see below). In this way the WTD algorithm provides a measure of taxonomic disagreement between the observed and expected taxonomic information on a per-pathway basis.

Finally, there is a detail to discuss about the symmetric nature of the WTD. Intuitively the WTD distance captures the approximate taxonomic agreement between two positions on the NCBI Taxonomy Database. However, the distance is symmetric to the with regard to the lineage of the observed and expected taxa, making it impossible to tell from distance value alone if the expected taxon is *in the lineage of* the observed taxon (i.e., the observed taxon is a descendant of the expected taxon) or otherwise (i.e., the two taxa have diverged). This is resolved by calculating the WTD relative to expected taxon position. Distances calculated on a path where the expected taxon *is in the lineage* of the observed taxon are given a non-negative value and are negative otherwise. Intuitively, non-negative distances represent the degree of specificity of an observed LCA taxon within the descendants a taxonomic range, while large negative distances represent how much disagreement exists between the two positions. In the case of multiple taxonomic ranges, we have a preference for the closest taxonomic range that is still positive. Thus, the WTD algorithm first attempts to return the minimum non-negative distance, and if no positive distance is found than the maximum negative distance (i.e., closest to zero) is returned.

Calculation

We will now describe the details of the weighted taxonomic distance algorithm, $computeWTD$, which can be applied to a set of predicted pathways from an ePGDB. Globally, the algorithm will provide a distance value for each predicted pathway, expressing the taxonomic disagreement between the observed and expected taxonomic signal. Lets first provide some notation for pathways, reactions, and associated CDS annotations in a ePGDB. For any ePGDB $\mathcal{G}$, we introduce the following notations for convenience. The notation $P(\mathcal{G})$ denotes the list of base pathways in $\mathcal{G}$. Next $R(p,\mathcal{G})$ denotes the set of reactions in base pathway p which appears in $\mathcal{G}$. Finally, $CDS(r,\mathcal{G})$ denotes the list of CDS annotations associated with reaction/enzyme r in $\mathcal{G}$. The observed taxon for each pathway is obtained in the following way. For a given pathway p and ePGDB $\mathcal{G}$, the observed taxon $p^{(O)}$ is calculated by the $pathwayObservedTaxon$ procedure below. The procedure collects all taxonomic CDS annotations for associated with each reaction r in pathway p, and calculates and returns the lowest common ancestor as the observed taxonomy $p^{(O)}$ via the LCA algorithm described above.

procedure *pathwayObservedTaxon*

input: $p, \mathcal{G}$ /* where p is a base pathway in ePGDB $\mathcal{G}$ */

$T \leftarrow \emptyset$ /* list of taxa, initially empty */

1 **foreach** r in $R(p, \mathcal{G})$

2 **foreach** c in $CDS(r, \mathcal{G})$

/* where t_c is the taxa annotation from refseq */

3 $T \leftarrow T \cup \{t_c\}$

4 **endfor**

5 **endfor**

6 **output** $\boldsymbol{p}^{(\boldsymbol{O})} \leftarrow LCA(T)$

Algorithm 1. The procedure *pathwayObservedTaxon* computes the observed taxon for any pathway p present in ePGDB $\mathcal{G}$. The algorithm collects for pathway p the associated list of CDS annotations (lines 2-4), for each reaction (line 1-5), in the set variable T. Next it applies the LCA [23] algorithm on the set of taxa T to determine the lowest common ancestor, as the observed taxon $p^{(O)}$ for pathway p.

Next, we compare the observed taxon $p^{(O)}$ (i.e., a taxon in the NCBI Tree) against the set of expected MetaCyc pathway taxonomic range(s). Note that for a base pathway p, in the context of the ePGDB under consideration $\mathcal{G}$, we denote its non-empty set of taxa in the MetaCyc taxonomic range by $TR^{(MetaCyc)}(p)$, and use $p^{(O)}$ to denote the observed taxon computed by algorithm *pathwayObservedTaxon*. Also, recall for a taxon t we denote its lineage $L(t)$. Note that since the MetaCyc taxonomic range is a curated set of taxonomic information associated with a pathway, we would like to adjust the WTD to reflect if the expected taxonomy t is *in the lineage of* observed taxonomy $p^{(O)}$. Here the sign of the WTD is used to reflect the position of $p^{(O)}$ relative to t: non-negative if taxonomy t is *in the lineage of* observed taxonomy $p^{(O)}$ and negative otherwise. The pairwise distance is calculated between each taxon in $TR^{(MetaCyc)}(p)$ and $p^{(O)}$, non-negative distances are added to C_p, and negative distances are added to C_n. The WTD is computed with a preference to non-negative distances. If C_p is not empty, the minimum of the non-negative distances C_p is returned. If C_p is empty, the maximum of the negative distances C_n is returned.

procedure computeWTD

input: $p, \mathcal{G}$ /* where p is a base pathway in ePGDB $\mathcal{G}$ */

$C_p \leftarrow \emptyset$ /* set of non-negative distances, initially empty */

$C_n \leftarrow \emptyset$ /* set of negative distances, initially empty */

1 $p^{(O)} \leftarrow pathwayObservedTaxon(p)$ /* observed LCA-based taxon */

2 **foreach** t in $TR^{(MetaCyc)}(p)$

3 **if** $t \in \mathrm{L}(p^{(O)})$ /* expected taxon in lineage of observed */

4 $C_p \leftarrow C_p \cup \{D(t, p^{(O)})\}$

5 **else**

6 $l \leftarrow LCA(t, p^{(O)})$

7 $C_n \leftarrow C_n \cup \{-\left(D(l,t) + D\left(l, p^{(O)}\right)\right)\}$

8 **endfor**

9 **if** $C_p \neq \emptyset$ /* positive distance set not empty */

10 **output** **min** $(\boldsymbol{C_p})$

11 **else** /* use negative distance */

12 **output** **max** $(\boldsymbol{C_n})$

Algorithm 2. The procedure *computeWTD* computes the WTD for a given pathway p. The taxonomic assignment $p^{(O)}$, generated via $pathwayObservedTaxon(p)$ (line 1), is compared to the non-empty set of taxa from the MetaCyc taxonomic range for pathway p MetaCyc $TR^{(MetaCyc)}(p)$ (lines 2-8). In line 3, if taxonomic range taxon t is *in the lineage of* $p^{(O)}$ then the distance is non-negative, is calculated as $D(t, p^{(O)})$, and added to the set of non-negative distances C_p. If the condition in line 3 does not hold, then t is not *in the linage* of $p^{(O)}$ and therefore t and $p^{(O)}$ *diverge*, and thus have a LCA. The distance is calculated as the sum of the two paths to this LCA (lines 5-7), made negative to show divergence, and added to the set of negative distances C_n. Finally, if set of non-negative distances C_p is not empty, the minimum C_p is output (lines 9-10), otherwise the maximum of the set of negative distances C_n (lines 11-12).

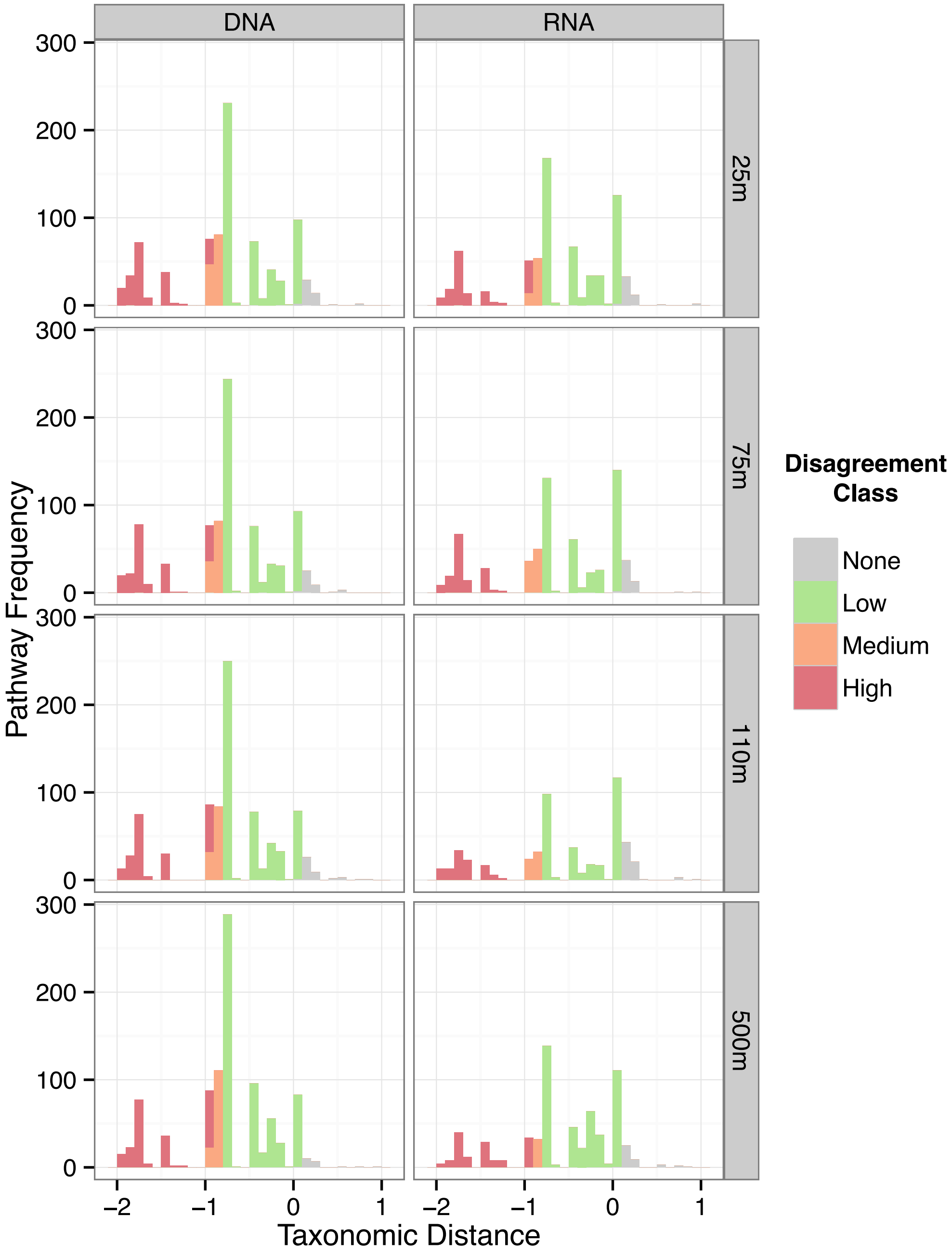

Figure S2. Distribution of Weighted Taxonomic Distance from HOT Predicted Pathways. Positive distances represent instances where the observed taxonomy was a descendant of the MetaCyc taxonomic range for that pathway, while negative distances represent divergent taxonomies. Predicted pathways were classified into taxonomic disagreement classes based on the weighted distance distribution for the sample; "None" contains positive distances, "Low" contains the upper two negative quartiles, while the "Medium" and "High" disagreement classes contain the lower two negative quartiles, respectively.

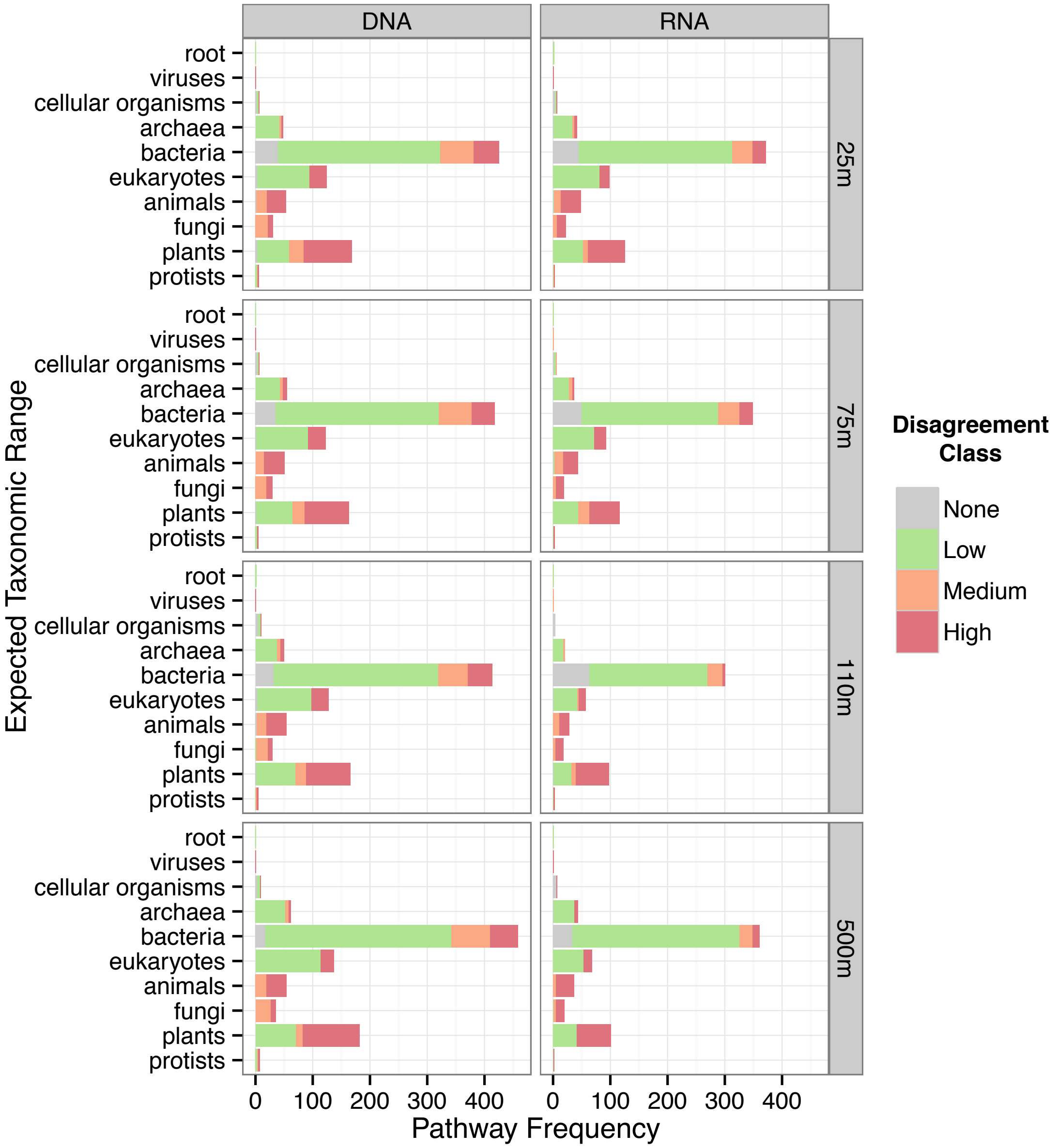

Figure S3. Disagreement class distribution of HOT predicted pathways by expected taxonomic range. Predicted pathways from HOT datasets were classified into their disagreement class based on WTD. Tabulating these pathways by expected taxonomic range the majority of pathways classified in the "Medium" and "High" disagreement classes have expected taxonomic ranges within "animals", "fungi", and "plants".

Table S10. Total predicted pathways for pairwise combined tier-2 BioCyc genomes: *Aurantimonas manganoxydans SI85-9A* (A), *Bacillus subtilis subtilis 168* (B), *Caulobacter crescentus NA1000* (C), and *Helicobacter pylori 26695* (H).

	A	B	C	H
A	394	497	424	435
B		361	481	402
C			378	416
H				210

Table S11. Number of candidate pathways that are potentially distributed by set-difference calculation (full listing in **Additional File 2**).

	A	B	C	H
A	-	4	1	6
B		-	6	11
C			-	2
H				-

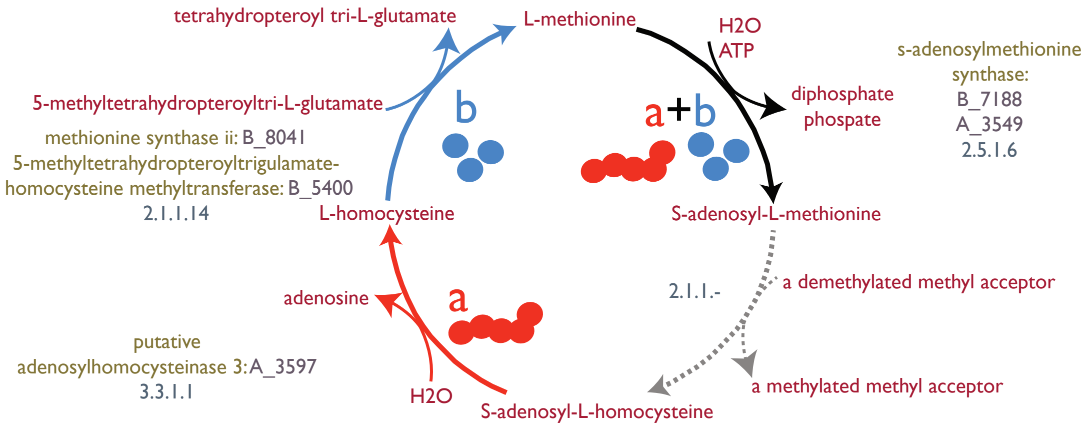

Figure S4. An example of a plausible emergent metabolism pattern. The completion of the pathway requires participation from multiple taxa, e.g., *Aurantimonas manganoxydans SI85-9A* (a) and *Bacillus subtilis subtilis 168* (b). Pathway glyphs produced by Pathway Tools can be supplemented with taxonomic information to enable the discovery of patterns of inter-pathway complementarity and potentially distributed metabolic pathways.

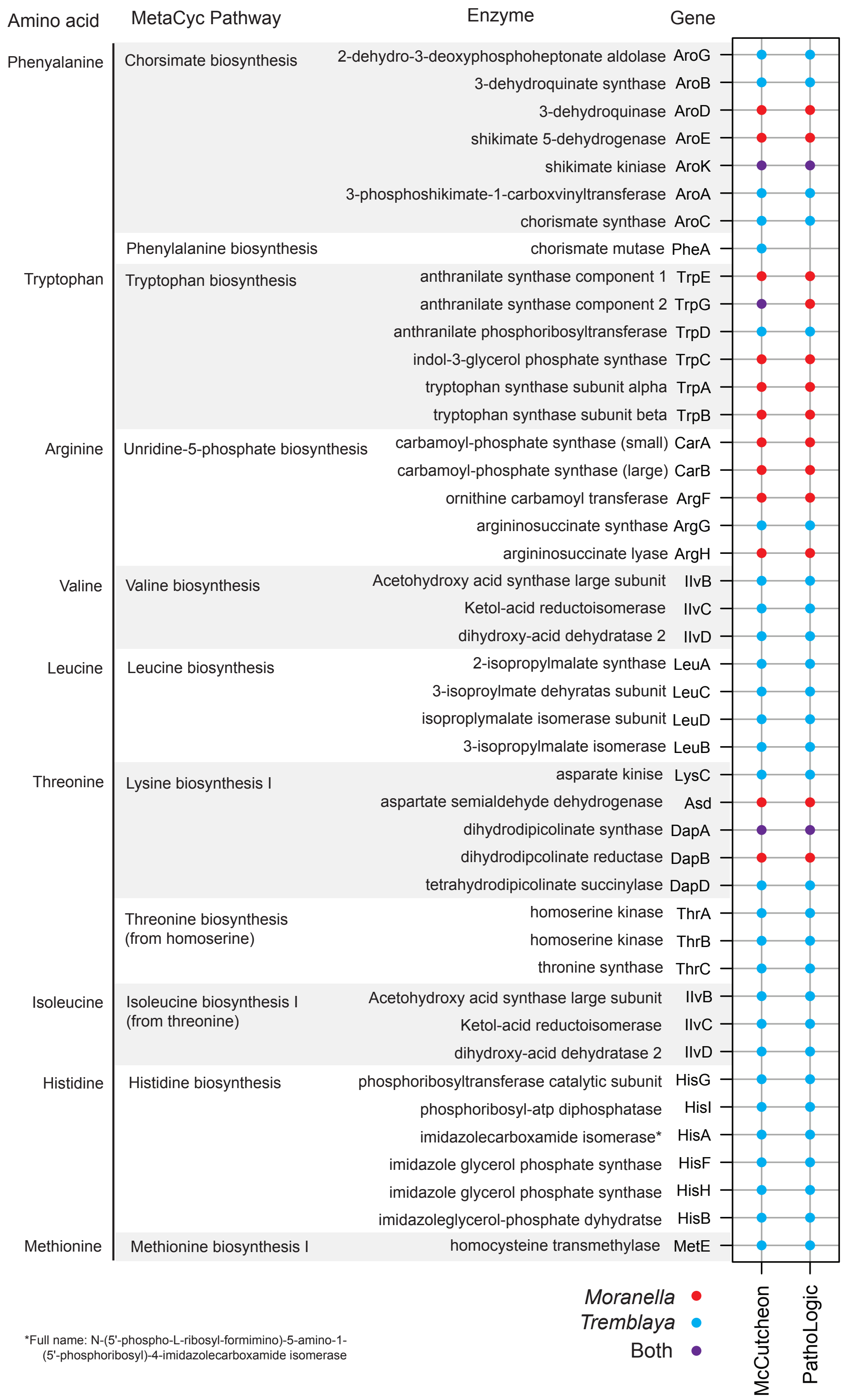

Figure S5. Comparison of predicted amino acid pathways in the *Candidatus Moranella endobia* and *Candidatus Tremblaya princeps* genomes. Dots represent detected presence of the pathway enzymes in *Moranella* (red), *Tremblaya* (blue), or both genomes (purple).

Table S12. Summary statistics of pathway prediction for the HOT metagenome and metatranscriptome.

Sample	GenBank SRA	Size (bp)	Reads	ORFs	Annotated CDS	MetaCyc Reactions	Predicted Pathways
HOT 25m	SRX007372	160,254,663	623,559	405,613	214,149	4,138	864
HOT 75m	SRX007369	164,376,456	673,674	430,689	222,572	4,052	854
HOT 110m	SRX007370	127,754,820	473,166	336,035	165,775	4,133	860
HOT 500m	SRX007371	274,826,172	995,747	714,743	361,193	4,464	949
HOT 25m (cDNA)	SRX016893	139,331,608	561,821	234,404	85,781	3,433	723
HOT 75m (cDNA)	SRX016897	133,294,602	557,718	203,359	66,855	3,208	669
HOT 110m (cDNA)	SRX156384	90,843,408	398,436	135,107	36,912	2,549	532
HOT 500m (cDNA)	SRX156385	127,589,826	479,661	207,465	71,400	3,034	641

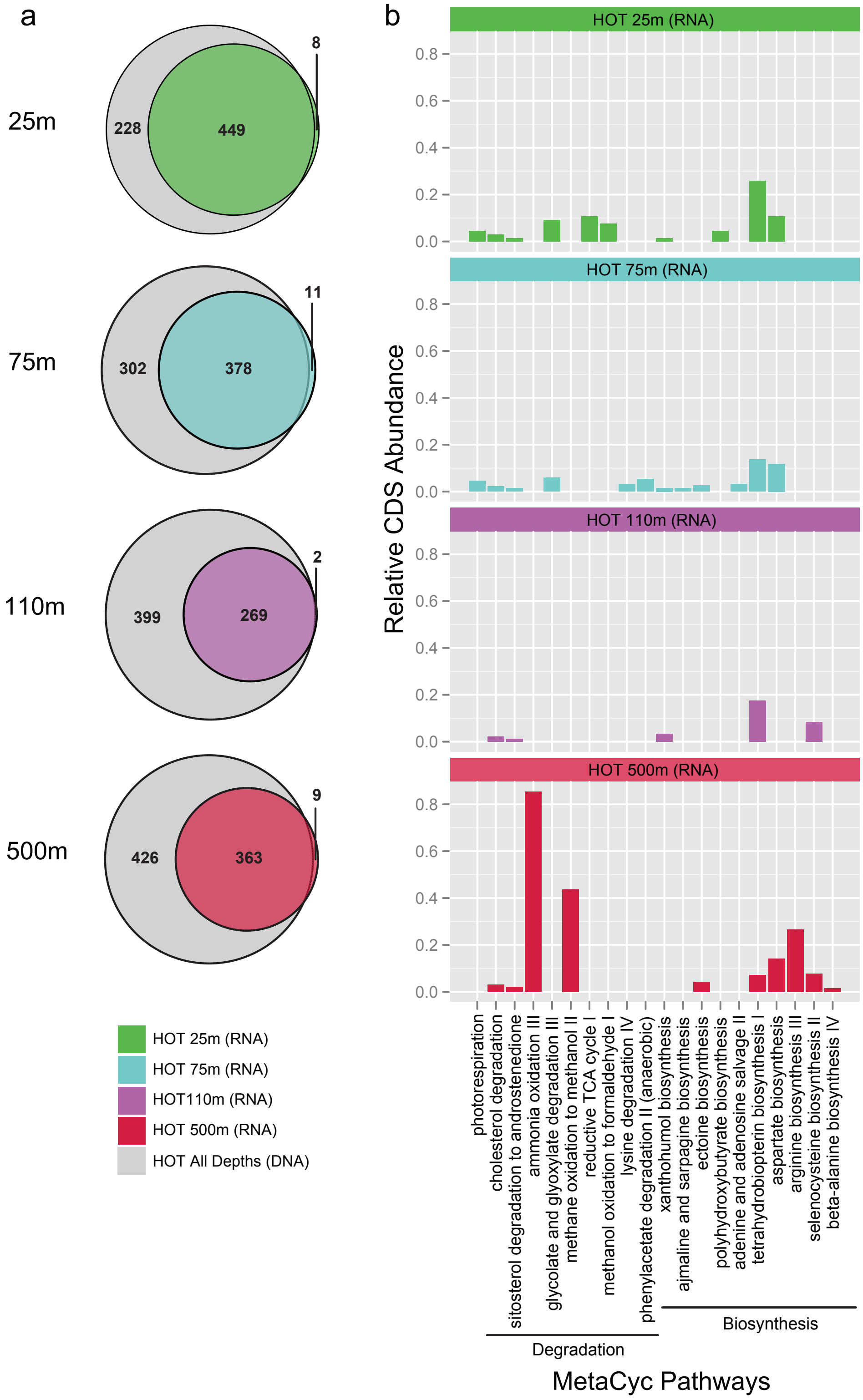

Figure S6. Overview of unique transcriptomic signal. **(a)** Euler diagrams comparing common genomic and transcriptomic pathways for each depth. **(b)** Unique transcriptomic pathways projected to all depths.

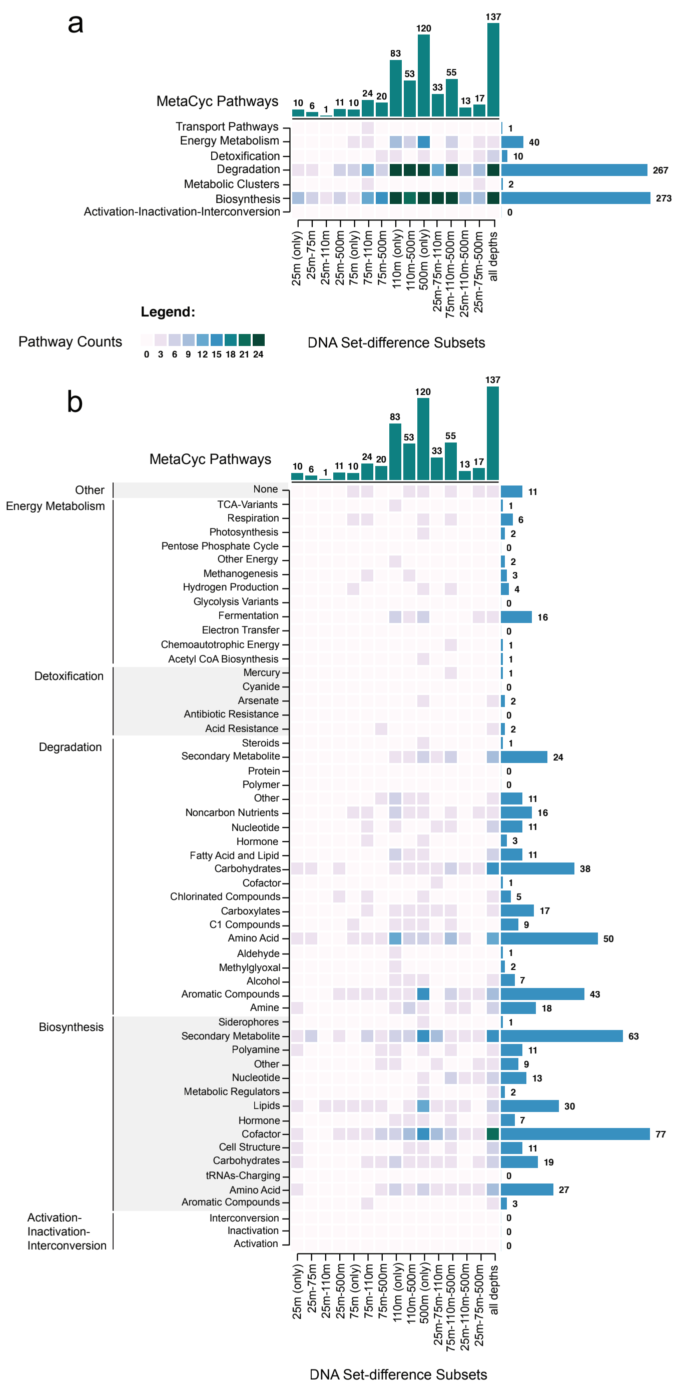

Figure S7. Predicted pathways predicted unique to DNA samples. **(a)** Unique DNA pathways projected at the highest MetaCyc classification. **(b)** Unique DNA pathways projected to the next MetaCyc sub-classification.

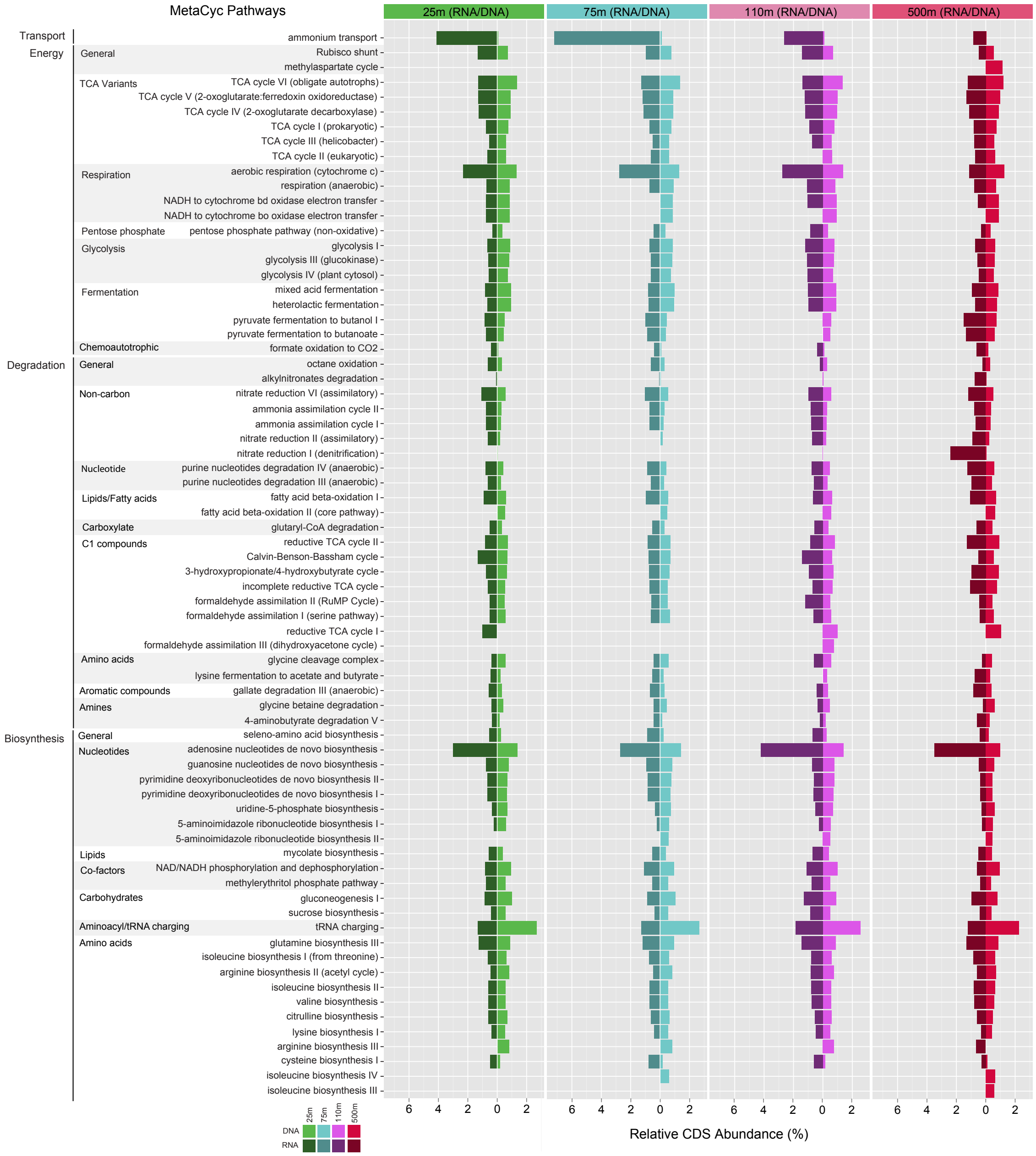

Figure S8. Top-40 predicted pathways based on coding DNA sequence (CDS) and transcript abundance from four HOT depth intervals. The most abundant pathways were largely stable between samples, with the Rubisco shunt, pyruvate fermentation, NADH to cytochrome electron transfer, aerobic respiration, nitrate reduction, and pyruvate fermentation varying between sunlit and dark ocean waters.

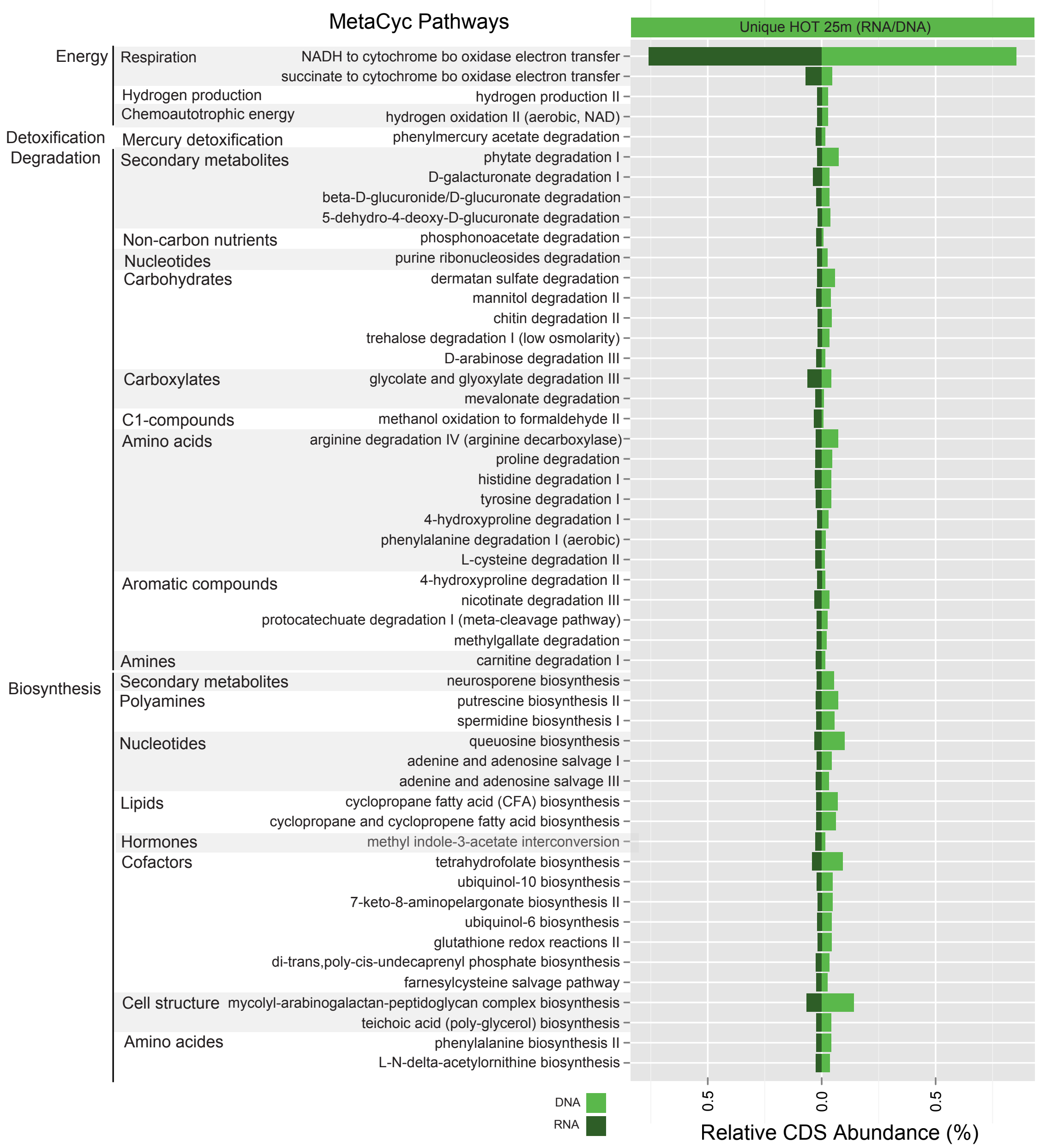

Figure S9. Genomic and transcriptomic signal for pathways unique to the sunlit surface (25 m) depth interval. The predicted presence of cytocrome oxidase electron transfer and reversible hydrogen production and oxidation indicate a strong signal for aerobic growth.

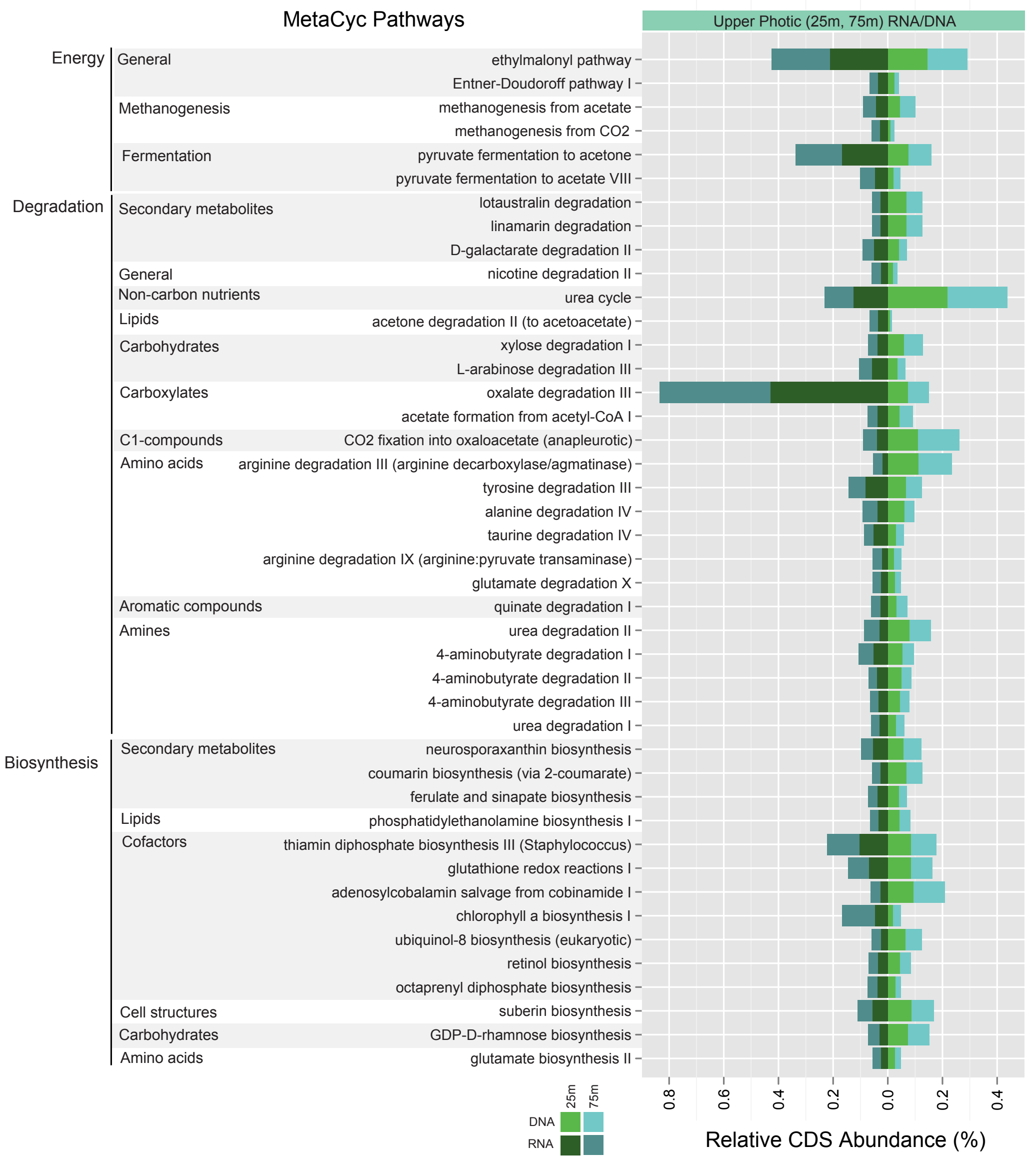

Figure S10. Genomic and transcriptomic signal for pathways unique to upper photic zone(25 m and 75 m) depth intervals. Notable pathways included chlorophyll a biosynthesis, the ethylmalonyl, Entner-Doudoroff, and pyruvate fermentation pathways.

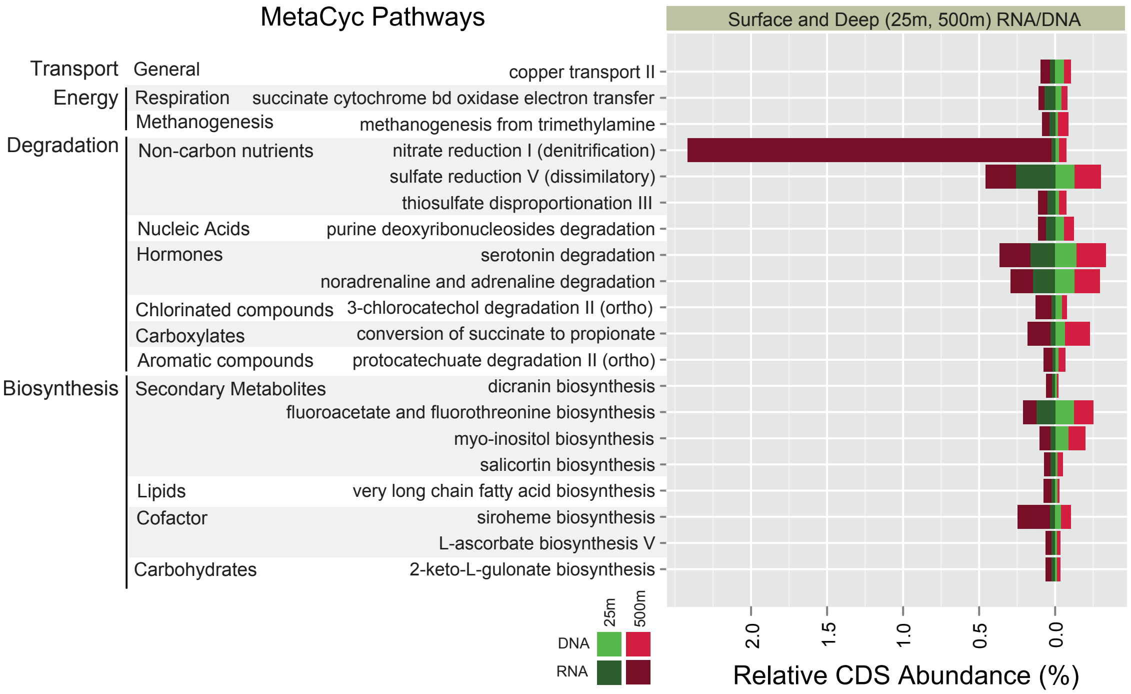

Figure S11. Genomic and transcriptomic signal for pathways unique to surface (25 m) and deep (500 m) depth intervals. There exist a limited number of pathways common to the surface and deep, but note that the largest signal is for nitrate and sulfate reduction, the first steps of sulfur recycling being shared with nitrate reduction.

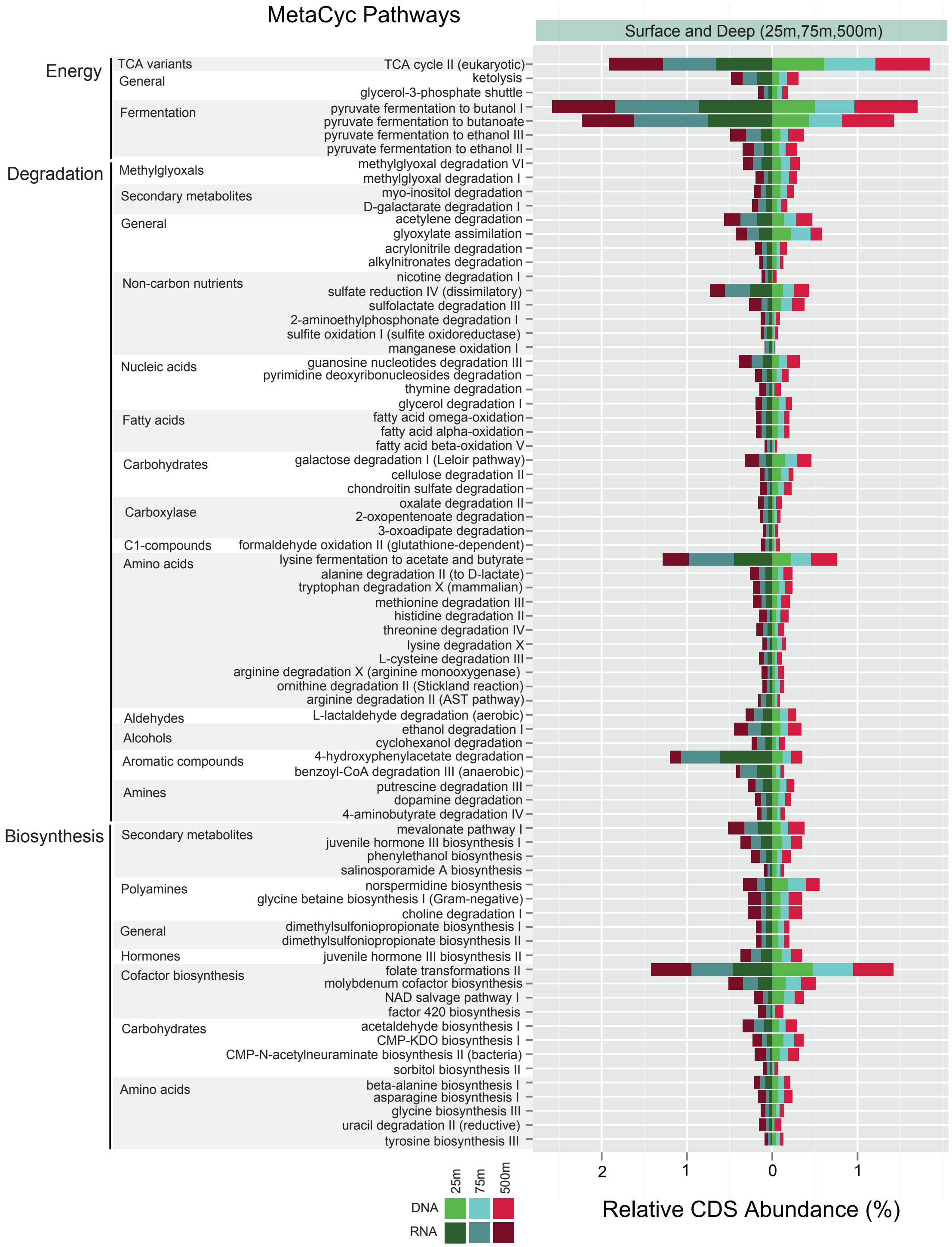

Figure S12. Unique pathways to the 'photic and deep' samples (25 m, 75 m, and 500 m). This set is characterized by a strong signal for the TCA cycle, ketolysis and pyruvate fermentation, as well as a large number of organic matter degradation pathways.

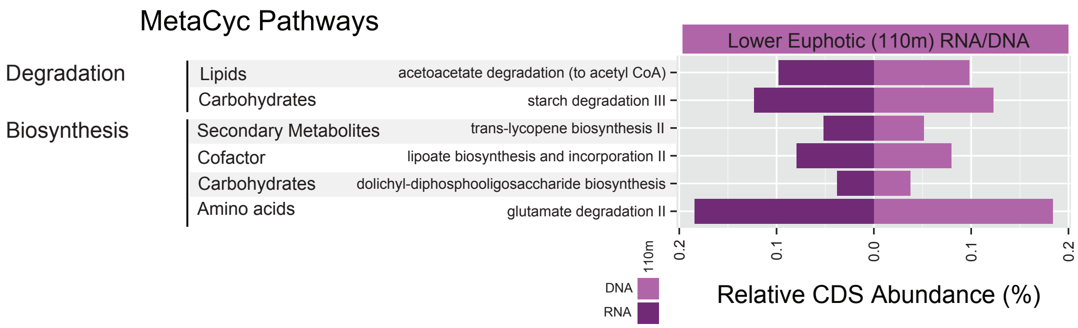

Figure S13. Unique pathways to the lower euphotic 110m sample.

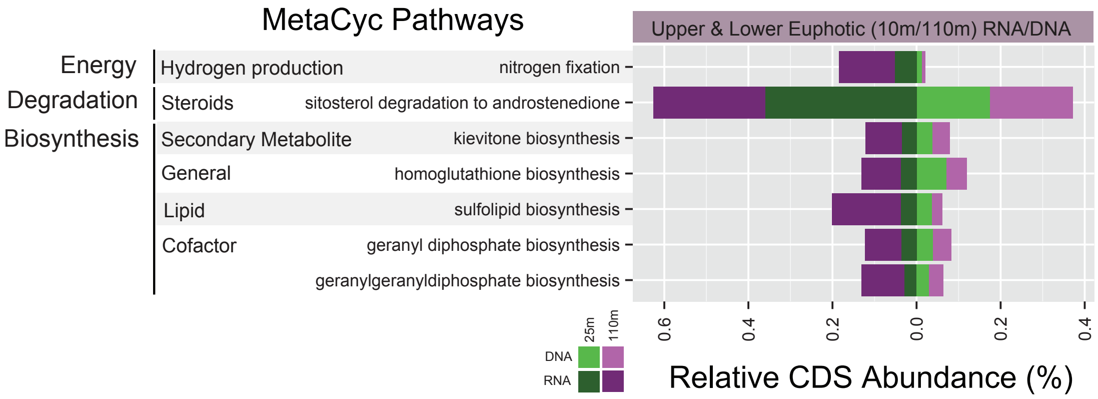

Figure S14. Unique pathways to the upper and lower euphotic 25m and 110m samples.

Table S13. Examples of observed pathway prediction hazards from the HOT analysis.

Pathway Name	EC Promiscuity	Pathway Variants	Unique Reactions	Taxonomic Range
threonine degradation IV	X			
lysine degradation X	X	X		
ethanol degradation	X			
molybdenum cofactor biosynthesis	X			
thiamin diphosphate biosyntheis		X		
adenosylcobalamin biosynthesis		X		
TCA cycle variants		X		
NAD-related pathways		X		
intra-aerobic nitrate reduction		X		
ammonia assimilation		X		
sucrose degradation II		X	X	
nitrate reduction		X	X	
dTDP-D-desosamine biosynthesis			X	
mannitol degradation I			X	
linamarin degradation				X
limonene degradation II (L-limonene)				X
